# Cortical oscillations and entrainment in speech processing during working memory load

**DOI:** 10.1111/ejn.13855

**Published:** 2018-02-16

**Authors:** Jens Hjortkjær, Jonatan Märcher‐Rørsted, Søren A. Fuglsang, Torsten Dau

**Affiliations:** ^1^ Hearing Systems Group Department of Electrical Engineering Technical University of Denmark Ørsteds Plads, Building 352 Kgs. Lyngby Denmark; ^2^ Danish Research Centre for Magnetic Resonance Centre for Functional and Diagnostic Imaging and Research Copenhagen University Hospital Hvidovre Hvidovre Denmark

**Keywords:** alpha and theta oscillations, EEG, n‐back task, pupillometry, speech entrainment

## Abstract

Neuronal oscillations are thought to play an important role in working memory (WM) and speech processing. Listening to speech in real‐life situations is often cognitively demanding but it is unknown whether WM load influences how auditory cortical activity synchronizes to speech features. Here, we developed an auditory n‐back paradigm to investigate cortical entrainment to speech envelope fluctuations under different degrees of WM load. We measured the electroencephalogram, pupil dilations and behavioural performance from 22 subjects listening to continuous speech with an embedded n‐back task. The speech stimuli consisted of long spoken number sequences created to match natural speech in terms of sentence intonation, syllabic rate and phonetic content. To burden different WM functions during speech processing, listeners performed an n‐back task on the speech sequences in different levels of background noise. Increasing WM load at higher n‐back levels was associated with a decrease in posterior alpha power as well as increased pupil dilations. Frontal theta power increased at the start of the trial and increased additionally with higher n‐back level. The observed alpha–theta power changes are consistent with visual n‐back paradigms suggesting general oscillatory correlates of WM processing load. Speech entrainment was measured as a linear mapping between the envelope of the speech signal and low‐frequency cortical activity (< 13 Hz). We found that increases in both types of WM load (background noise and n‐back level) decreased cortical speech envelope entrainment. Although entrainment persisted under high load, our results suggest a top‐down influence of WM processing on cortical speech entrainment.

## Graphical abstract

We used an auditory n‐back task to asses the effect of working memory (WM) load on cortical speech entrainment. Consistent with visual n‐back tasks, posterior alpha band power (8–13 Hz) decreased while frontal theta power (4–7 Hz) increased with WM load. At the same time, auditory cortical entrainment (< 13 Hz) to the envelope of the n‐back speech stimulus was found to decrease both with load level and with acoustic noise interference.

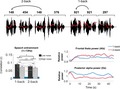

## Introduction

Cortical oscillations have been hypothesized to play a functional role in speech processing (Ghitza, 2011; Giraud & Poeppel, 2012). Oscillatory activity, particularly in the delta (1–3 Hz) and theta (4–7 Hz) frequency bands, has been found to entrain to the slow temporal modulations inherent in natural speech signals (Ahissar *et al*., 2001; Luo & Poeppel, 2007; Di Liberto *et al*., 2015). Selective attention is known to modulate this response by enhancing the entrainment between low‐frequency cortical activity and the speech stream that the listener is attending to relative to the ignored stream (Ding & Simon, 2012; Zion Golumbic *et al*., 2013; O'Sullivan *et al*., 2014). However, listening to speech in everyday life also involves working memory (WM) to maintain and relate speech content over time or to inhibit irrelevant information. Across modalities, WM tasks have been associated with different oscillatory networks in cortex (Roux & Uhlhaas, 2014), but potential relations to speech processing are unclear. Oscillatory power in higher‐order cortical areas are thought to influence speech‐entrained activity in auditory cortex (Park *et al*., 2015; Keitel *et al*., 2017), but it is unclear whether such functional couplings might reflect an interaction between WM processes and auditory processing of the speech stimulus.

The nature of a potential relationship between WM tasks and speech entrainment is not clear. Several scenarios are possible. First, although speech entrainment is known to be shaped by selective attention (Ding & Simon, 2012; Mesgarani & Chang, 2012; O'Sullivan *et al*., 2014), theta and alpha signatures of WM demands could reflect general WM processes that do not interact with auditory processing. In this case, attending to a speech stimulus is sufficient to establish an entrained response and additional task demands leave the entrainment response unaffected. Alternatively, higher degrees of WM load may distribute neural resources away from sensory processing of the speech stimulus and towards processing related to the cognitive task. Cortical responses evoked by visual stimuli during WM tasks have consistently been found to be attenuated with increasing cognitive demands (Gevins *et al*., 1996; Watter *et al*., 2001; Pratt *et al*., 2011; Scharinger *et al*., 2015, 2017). If this generalizes to speech entrainment, then higher WM load might be associated with a decrease in entrainment. Finally, it is also conceivable that increased task engagement associated with higher WM load may recruit additional neural resources for the processing of the task‐relevant stimulus. In this case, WM load would instead increase the cortical entrainment to the speech signal.

Numerous human electroencephalogram (EEG)/magnetoencephalogram (MEG) studies have related WM demands to changes in oscillatory power, particularly in the theta and alpha frequency ranges (Klimesch, 1999). Despite the consistent involvement of theta and alpha oscillations, the functional characterization of these oscillations in terms of specific WM functions is still debated. The n‐back task is often used to probe WM function (Owen *et al*., 2005). In an n‐back task, subjects are asked to detect whether the presented stimulus in a sequential stream of items matches the one presented n positions back. In visual n‐back tasks, increasing WM processing load (higher n) is associated with a frontocentral increase in theta power and a decrease in alpha band power at posterior recording sites (Gevins *et al*., 1997; Gevins & Smith, 2000; Pesonen *et al*., 2007; Haegens *et al*., 2014; Scharinger *et al*., 2015, 2017). In tasks involving memorization of a number of items (e.g. the Sternberg task), on the other hand, both alpha band power and theta band power have been found to increase with the number of elements held in memory (Krause *et al*., 1996; Raghavachari *et al*., 2001; Jensen & Tesche, 2002; Jensen *et al*., 2002; Leiberg *et al*., 2006; Obleser *et al*., 2012).

Different WM processes are thus associated with different and sometimes opposing alpha–theta changes. In a minimal definition, WM involves a temporary memory storage (sensory buffers) and attention‐related control functions for maintenance and manipulation of WM content (‘central executive’) (Baddeley, 2003). Executive functions have been further divided into memory *updating* functions that actively maintain and replace information, and WM *inhibition* that suppresses information that is not relevant to the current task (Miyake *et al*., 2000). The n‐back task has been suggested to specifically target WM updating load (Miyake *et al*., 2000; Scharinger *et al*., 2015). In visual tasks, inhibitory demands on WM are often manipulated with incongruent items, for example in a flanker task. Although updating load has been related to decreases in alpha power, inhibitory WM load has been associated with increasing alpha power (Snyder & Foxe, 2010; Händel *et al*., 2011), consistent with the notion of alpha oscillations as a suppression mechanism (Jensen & Mazaheri, 2010; Foxe & Snyder, 2011). In auditory tasks, acoustic degradations or noise is a common source of interference and has been shown to increase behavioural WM load (Pichora‐Fuller *et al*., 1995). For spoken or memorized words, acoustic degradations have been associated with increasing alpha power at posterior channels (Obleser *et al*., 2012; Wöstmann *et al*., 2017), consistent with an increase in inhibitory WM load. In natural speech processing, however, executive functions related to the maintenance of relevant information and the inhibition of irrelevant information are typically engaged at the same time. Yet, it is unclear how these WM processes may interact in speech perception. Multiple studies have reported that WM load influences the ability to ignore distracting information, but the nature of this relation appears to be highly dependent on the stimulus type and the type of cognitive task involved (Lavie *et al*., 2004; San Miguel *et al*., 2008; Sörqvist *et al*., 2012; Vandierendonck, 2014; Scharinger *et al*., 2015).

Recent studies indicate that speech‐entrained activity in the auditory cortex is functionally dependent on oscillatory power in multiple frontoparietal networks (Park *et al*., 2015; Keitel *et al*., 2017). Keitel *et al*. (2017) recently reported that entrained auditory cortical activity, quantified as the mutual information between the phase of low‐frequency activity in auditory cortex and the phase of slow speech envelope modulations, interacted with oscillatory power in distinct cortical networks. In particular, delta entrainment in the auditory cortex was dependent on central alpha and frontal beta power and modulated parietal theta power. This could indicate a top‐down influence on speech‐entrained activity in auditory cortex by oscillations within a larger cortical network involved in cognitive control or attention. Such a top‐down influence could reflect language‐specific functions such as semantic memory (Keitel *et al*., 2017), but could also be related to more general WM functions. To test more directly whether WM processing influences cortical speech entrainment, however, it needs to be demonstrated that imposing a WM processing load in behavioural tasks influences concurrent speech entrainment.

Here, we developed an experimental paradigm to investigate influences of WM load on cortical speech envelope entrainment. We designed a ‘number speech’ material consisting of sequences of spoken numbers that match important properties of natural continuous speech. During speech listening, participants performed either a 1‐back or 2‐back task with the speech sequences embedded in either a high or a low level of background noise. This allowed us to examine the individual and combined effects of WM updating (n‐back level) and inhibition (noise level) load during continuous speech processing. We recorded the EEG as well as changes in pupil sizes which are often used as a physiological marker of WM demands (Van Gerven *et al*., 2004; Zekveld *et al*., 2010; Scharinger *et al*., 2015; Wendt *et al*., 2016). To examine potential differences in speech entrainment during the different load conditions, we used regression techniques to analyse the relationship between ongoing low‐frequency cortical activity and envelope fluctuations in the corresponding speech signal (Lalor *et al*., 2009; Ding & Simon, 2012). Using continuous speech, our paradigm also allowed us to study the dynamics of prolonged WM load and load‐related measures over longer time segments.

## Materials and methods

### Participants

Twenty‐two healthy volunteers (six females, aged 19–28, mean age: 24, SD: 3 years) participated with informed consent. Eye‐tracking data were recorded in 15 of the participants. All participants reported normal hearing. The experiment was approved by the Science Ethics Committee for the Capital Region of Denmark (protocol no. H‐16036391) and conducted in accordance with the Declaration of Helsinki.

### Speech stimuli

**Figure 1:**
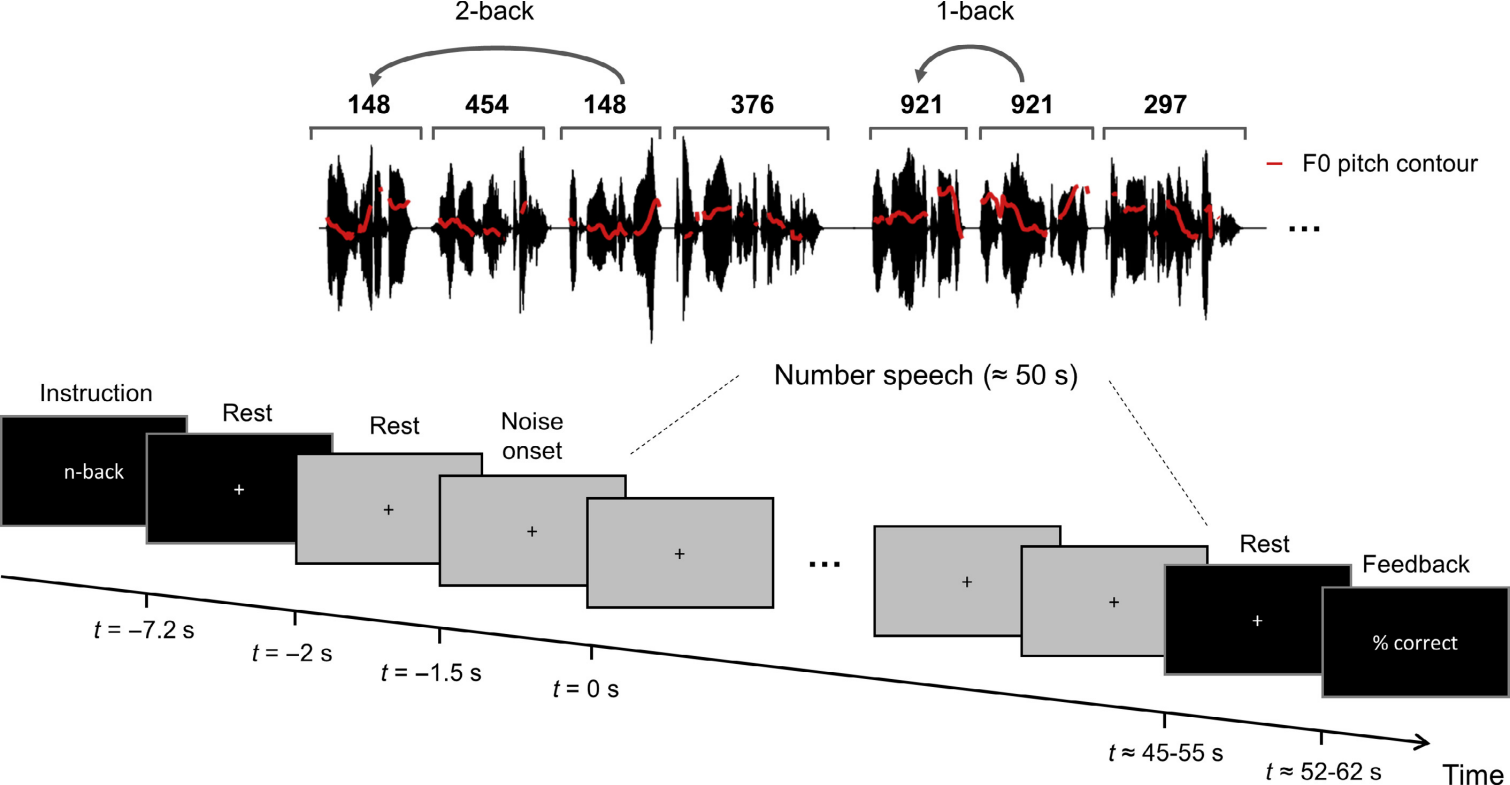
Schematic illustration of the trial structure and task. Electroencephalogram and pupillometry were recorded, while subjects listened to continuous speech stimuli consisting of spoken number sequences. Red lines on the waveform represent the pitch contour of the continuous speech signal. In different trials, listeners identified either 1‐back or 2‐back number targets in different levels of background noise. Please see the Methods section for details.

We created a speech material that could be used to control the WM load imposed on the listener and monitor their task performance during listening. The speech material consisted of spoken number sequences created to match natural continuous speech in terms of syllabic rate, intonation and sentence rhythm. First, two‐ or three‐digit numbers were read by a male Danish speaker and recorded in an anechoic chamber. For each number, several tokens spoken in rising or falling intonation patterns were recorded. The recorded number tokens were afterwards concatenated into sequences of ‘number sentences’ consisting of three or four numbers (see Fig. 1). The time interval between numbers was set at random durations ranging between 150 and 230 ms, and the time interval between number sentences was set randomly between 300 and 700 ms to match the word and sentence rhythm of natural speech. The number sentences were then used to synthesize long sequences of spoken numbers for the experimental trials. We created 20 trial lists each of 30 spoken numbers (resulting in durations between 45 and 55 seconds). Each trial list contained *n* = 1, 2, 3 back repetition targets, that is numbers which were identical to the number presented *n* numbers previously. We ensured that the n‐back targets were equally distributed between the first and second half of the list.

To generate speech‐shaped stationary background noise with the same spectral characteristics as the original speech stimuli, we computed the average of a large number of speech waveforms until the signal had no distinct slow envelope modulations. In the experiment, we wanted to impose the noise at a signal‐to‐noise ratio (SNR) that resulted in maximal interference without disrupting speech intelligibility. For this reason, we measured speech reception thresholds for the number tokens in a separate psychoacoustic test with four normal‐hearing listeners not participating in the main experiment. The lowest SNR point on the psychometric function that resulted in 100% correct identification was estimated to 0 dB SNR. In the main experiment, this noise level was defined as the ‘high‐noise condition’. A noise level 10 dB lower (i.e. 10 dB SNR) was defined as the ‘low‐noise condition’. Different speech‐shaped noise tokens were used in every trial, that is the noise was not frozen.

For the analysis of speech entrainment, the temporal amplitude envelopes of the continuous speech signals were extracted using an auditory model of envelope processing in the peripheral auditory system. The audio waveforms were first passed through a gammatone filterbank mimicking the spectral filtering characteristics of the basilar membrane (Patterson *et al*., 1987). At the output of each filter, the envelope was extracted via the Hilbert transform and raised to the power 0.3 to account for the compressive response of the inner ear (Plack *et al*., 2008). The spectrally decomposed envelopes were then resampled to match the EEG sampling rate and averaged across frequency channels.

### Experimental design

To control WM load during speech listening, participants listened to the continuous speech stimuli while performing an n‐back task in different levels of background noise. The conditions formed a 2 × 2 factorial design consisting of two n‐back task levels (1‐back, 2‐back) and two noise levels (low noise: 10 dB SNR, high noise: 0 dB SNR). In the 1‐back condition, participants were asked to detect whenever a number was repeated, and in the 2‐back task, they detected whether the presently spoken number was the same as one spoken two times back (Fig. 1). Note that repeated numbers were not acoustically identical but different speech tokens of the same number within the continuous speech stream. The same speech lists were used in the different n‐back conditions such that subjects heard the same speech stimuli in the two different behavioural contexts. As the occurrences of 1‐2‐3 back repetitions were equally distributed, the same occurrences acted as either targets or lures (repetitions to be ignored) depending on the n‐back task.

Figure 1 presents a schematic illustration of the trial timeline. Each trial began with a 7.2 s silent resting baseline where subjects fixated on a cross positioned in the middle of a black background screen; 2 s after the onset of the resting baseline, a green screen was shown for 200 ms to measure the pupil light reflex (not shown in Fig. 1), followed by another 5 s of a black screen baseline. Following the black screen baseline, a grey screen was presented for 500 ms before the onset of the sound stimulation. During sound stimulation, the participants maintained eye fixation on a cross on the grey background screen. The sound stimulation started with 1.5 s of the background noise at 0 dB or 10 dB SNR before the onset of the speech stimulus. During the following ~ 45–55 s presentation of the speech stimulus, the participants were asked to press a button when an n‐back target was detected. The participants were not instructed to use of any particular finger for responding. They were not informed about the noise level prior to the sound presentation. Responses were considered correct when they occurred between the onset of the target number and the onset of the following number plus an additional 200 ms. Responses that did not fall in this time interval were considered false alarms. After the speech task, the pre‐trial baseline and screen flash were repeated. Subjects performed eight initial training trials during which they received feedback whenever n‐back targets occurred in the speech stimulus. During the main experiment, feedback was only provided between trials by showing the average per cent correctly identified n‐back targets. Each participant performed 10 trials for each of the four experimental conditions. Lists contained either four (15 of 20 lists) or three (5 of 20 lists) n‐back targets.

### Data acquisition

The experiment was performed in an electrically shielded double‐walled sound booth (IAC Acoustics, North Aurora, IL, USA). The subjects were seated 60 cm in front of a presentation screen with dim background lighting that was kept constant for all participants. The auditory stimuli were presented via ER‐2 insert earphones (Etymotic Research, Elk Grove Village, IL, USA). The speech stimuli were presented at a fixed level of 65 dB SPL. The level of the speech stimuli was kept constant, and the level of background noise relative to the speech signal varied across noise conditions.

Electroencephalogram was recorded continuously at 64 scalp electrodes according to the international 10/20 system using a BioSemi ActiveTwo system (BioSemi, Amsterdam, Netherlands). The sampling rate was 512 Hz. Additional electrodes were placed on the left and right mastoids. Eye movements were detected using six bipolar electrooculographic channels positioned vertically and horizontally around the eyes.

For 15 subjects, pupil sizes were recorded using an Eyelink 1000 desktop system (SR Research Ltd., Ottawa, ON, Canada) with a sampling frequency of 250 Hz. Measurements were conducted on one eye, which varied between subjects. The eye‐tracking system was calibrated at the beginning of the experiment using a custom calibration routine.

### Data pre‐processing and analysis

#### Behavioural data

A measure of d‐prime (d’) was used to estimate subjects’ sensitivity in the n‐back task. This was defined as the difference between the inverse cumulative distribution function (CDF) of correct n‐back target detections (hits) and the inverse CDF responses made in the absence of a target (false alarms). To examine performance in the time course of the trial, we also computed the percentage correctly identified n‐back targets at their temporal positions in the trial.

#### EEG data pre‐processing

The EEG data were analysed using MATLAB and the FieldTrip toolbox (Oostenveld *et al*., 2011). The data were epoched from 5 s before the onset of the speech stimulus to 45 s after the speech onset. The data were high‐pass filtered at 0.5 Hz, re‐referenced to the average of the two mastoid electrodes and resampled to 128 Hz. For one subject, the data were re‐referenced to the average of all 64 scalp electrodes due to noisy mastoid electrodes. Bad (i.e. noisy) channels were identified visually and removed from the data. On average, 2.4 ± 1.9 channels were rejected. The bad channels were interpolated using a nearest neighbour method average.

The logistic infomax independent component analysis (ICA) algorithm (Bell & Sejnowski, 1995; Delorme & Makeig, 2004; Winkler *et al*., 2015) was used to decompose the re‐referenced EEG data from each subject high‐pass filtered at 1 Hz. The components were visually inspected, and artefactual components were rejected. On average, 6.9 ± 2.6% of the components were rejected (2–7 components). Most of the rejected components were considered electroocular (EOG) artefacts and were highly correlated with the EOG electrodes. The remaining components were identified as either muscle or cardiac‐related artefacts that appeared consistently across trials. The ICA‐derived mixing matrices were thereafter used to spatially filter out artefactual activity from the original EEG data high‐pass filtered at 0.5 Hz (Winkler *et al*., 2015). Trials were inspected visually for artefacts after ICA cleaning, and remaining bad trials were removed. Additionally, trials in which the subjects detected < 25% of the target were rejected from further analysis. On average, 7.6 ± 4.2 trials were rejected per subject. Three subjects with more than 50% of the data rejected in any given condition were removed from further analysis. For the remaining 19 subjects, there were no statistical differences in the number of trials removed between conditions (n‐back and noise interaction: *F*_1,18_ = 0.9404, *P *=* *0.345, n‐back: *F*_1,18_ = 0.0705, *P *=* *0.7937, noise: *F*_1,18_ = 1.8331, *P *=* *0.192). On average, 8.1 ± 1.4 trials remained in each condition for the remaining subjects.

We examined relative changes in theta band power and alpha band power between the experimental conditions. Theta activity was defined in the frequency range from 4 to 7 Hz and alpha from 8 to 13 Hz. Filtering was performed using high‐order finite impulse response filters. To compute band power, we calculated the sum of the squared absolute values of the filtered EEG signal for each of the frequency ranges in time windows of 5 s with 90% overlap. To account for individual differences, the power measures were normalized globally by dividing the power measures in each trial by the global average in band power across all trials. To further explore oscillatory power changes over a larger frequency range, we examined time–frequency representations (TFRs) of power changes by computing the spectral power as above but in 2‐Hz wide frequency analysis windows from 1 to 30 Hz, in steps of 0.5 Hz. The TFRs were normalized per frequency bin to the grand average power across all trials.

To study whether cortical EEG speech entrainment is modulated by working memory‐related processes, we derived temporal response functions (TRFs) (Lalor *et al*., 2009; Ding & Simon, 2012) that map linearly from the envelope of the continuous speech signal *S*(*t*) to the EEG responses *R*(*t*,*n*):

$$\hat{R}\left(t,n\right)\;=\;\sum_{l=1}^{L}h\left(\tau_{l},n\right)S\left(t-\tau_{l}\right)$$

where *n *=* *1…*N* denotes the number of electrodes and $\tau \;=\;\{\tau_{1},\tau_{2},\ldots \tau_{L}\}$ are the time lags between the stimulus and response. The TRFs, *h*(*τ*) were fitted separately on the data from each subject in each of the four experimental conditions. The TRFs were estimated using regularized regression with a quadratic penalty term (Lalor & Foxe, 2010). The regularization parameter was set to a fixed high value that gave the highest group‐mean leave‐one‐out prediction accuracy across all subjects (λ = 2^12^). The temporal response functions covered time lags ranging between 0 to 400 ms post‐stimulus in steps of 7.8 ms (sampling frequency of 128 Hz). The EEG data and speech envelopes were standardized to have zero mean and unit variance. The TRF models were computed using MATLAB code publicly available at http://www.ine-web.org/software/decoding.

For the TRF analyses, the EEG data were filtered between 1 and 13 Hz using high‐order finite impulse response filters. To quantify changes by either n‐back or noise on the TRF, the peak amplitude, as well as the latency of the peak, was examined. This was performed by extracting the maximum value of the TRF from 100 ms to 300 ms for each subject. The latency was defined as the time at which the peak value of the TRF occurred. A leave‐one‐trial‐out cross‐validation procedure was used to estimate model prediction accuracies in each experimental condition. The prediction accuracies were quantified as Pearson's correlation coefficient between the predicted EEG responses and the actual recorded EEG data on the held‐out trials. This correlation served as an indicator of the degree of speech entrainment, that is how tightly the cortical activity was synchronized to the speech envelope. We also examined band‐specific entrainment by filtering the EEG data in delta (1–3 Hz), theta (4–7 Hz) and alpha (8–13 Hz) ranges. In the statistical analysis of condition‐specific differences, we focused on 12 frontotemporal electrodes (FC5, FC3, FC1, FC2, FC4, FC6, F5, F3, F1, F2, F4, F6) previously found to be speech relevant (Di Liberto *et al*., 2015). To estimate chance‐level prediction, we used a permutation procedure where we predicted EEG responses based on the envelopes of nonmatching speech sequences. The 97.5% percentile of the chance distribution was defined as the noise floor.

#### Pupil data

Eye blinks were classified as samples in the time series where the absolute value of the pupil diameter exceeded three standard deviations of the mean pupil diameter. Blink‐corrupted segments were linearly interpolated from 350 ms before to 700 ms after the blink (Wendt *et al*., 2016). Trials containing more than 20% of corrupted data were rejected from further analysis. Furthermore, three subjects with more than 50% of rejected trials were excluded from the analysis. The subjects excluded due to noisy EEG data were not the same as the subjects excluded due to noisy pupillometry data. For the remaining subjects, 2 ± 3 trials were rejected. The blink‐removed data were smoothed using a 25‐point (100 ms) moving average filter. To account for individual differences between subjects, the data were normalized to the pupil diameter averaged over the 200 ms time window directly preceding the noise onset.

### Statistical analysis

We used repeated measures analyses of variance (ANOVA) to assess statistical group‐level differences between the 2 × 2 conditions (n‐back, noise) on all load‐related measures: behavioural performance, average pupil size and maximum pupil dilations, EEG band power, TRF peak amplitudes, TRF peak latencies and prediction accuracies. All statistical calculations were performed using MATLAB. Shapiro–Wilk tests (α = 0.05) were used to test for the normality assumptions of the parametric tests. For the analysis of the band‐specific oscillatory EEG power, we assessed group‐level differences in the time‐averaged theta band power over a frontal electrode (AFz) and alpha band power over a posterior electrode (Oz). This restriction was motivated by previous results showing effects of WM load in the theta band at frontal midline electrodes, as well as effects in the alpha band at posterior electrodes (Gevins *et al*., 1997; Gevins & Smith, 2000; Scharinger *et al*., 2015). To further explore differences in the trial‐averaged power across all electrodes sites, we performed cluster‐based permutation tests (as implemented in the Fieldtrip toolbox, Oostenveld *et al*., 2011). This procedure identifies spatially adjacent clusters of electrodes that show a significant power decrease or increase between the experimental conditions. Using *t*‐tests, we first computed the group‐level effects of n‐back and noise level on the trial‐averaged theta or alpha band power at all electrodes. In clusters with an electrode neighbourhood extent of 40 mm (on average 7.6 electrodes), the *t*‐statistic for electrodes exceeding a threshold of *P* < 0.01 (cluster alpha) was summed. To control for multiple comparisons, the maximum of the summed *t*‐statistic in the observed data was compared with a random partition formed by permuting the experiment condition labels (as implemented in *ft_freqstatistics*, Maris & Oostenveld, 2007). Clusters whose t‐statistic exceeded 99% (*P *<* *0.01) of the random partition were considered significant.

## Results

### Behavioural performance

**Figure 2:**
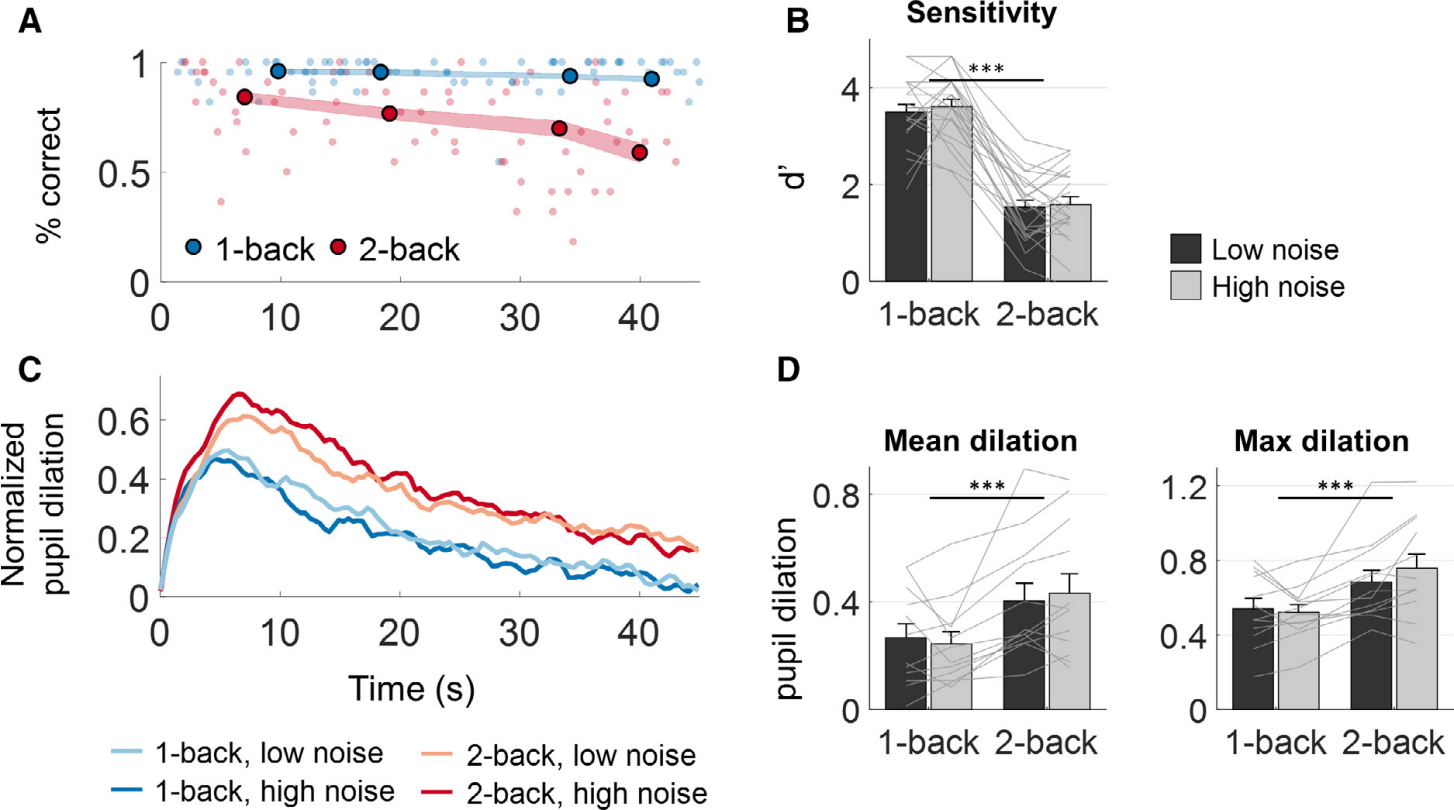
Behavioural performance (above) and pupil responses (below). (A) Percentage of correctly detected 1‐back and 2‐back targets during the speech trial. Larger circles represent the group average % correct at the average position of the targets. Shaded areas represent ± 1 SEM. (B) Behavioural sensitivity (d‐prime) for n‐back target detection measured over the trial duration. (C) The average trace of the pupil dilations relative to a pre‐stimulus baseline. (D) Mean and peak pupil dilation over the trial duration. Error bars represent ± 1 SEM ***P <* *0.001.

Response accuracy in the n‐back task (measured in d’) was significantly lower in the 2‐back condition compared to the 1‐back task (*F*_1,21_ = 203.77, *P *<* *0.001) but was not affected by the level of the background noise (*F*_1,21_ = 0.7487, *P *=* *0.397) (Fig. 2B). This result was expected as the higher noise level was predetermined to yield the speech fully intelligible. The n‐back targets (and lures) were uniformly distributed over the trial duration. This allowed us to inspect potential differences in response accuracy in different parts of the trials. As shown in Fig. 2A, the identification of 1‐back targets remained high throughout the trial, whereas the identification of 2‐back targets declined as the trial progressed.

### Influence of WM load on pupil dilations

All WM task conditions evoked a pupil dilation response with a peak 5–10 s after trial onset, followed by a gradual decrease in the remaining duration of the trial (Fig. 2C). The pupil dilations increased with the n‐back task level but did not increase additionally with the level of the background noise (Fig. 2D). Both the mean and peak pupil dilation were significantly higher for the 2‐back task compared to the 1‐back task (mean dilation, 0–45 s: *F*_1,11_ = 17.00, *P *=* *0.0017; peak dilation: *F*_1,11_ = 20.16, *P *<* *0.001). No significant effects of noise level were found on the pupil measures (mean dilation: *F*_1,11_ = 0.31, *P* = 0.58; peak dilation: *F*_1,11_ = 0.76, *P *=* *0.40).

### Influence of WM load on alpha and theta power

**Figure 3:**
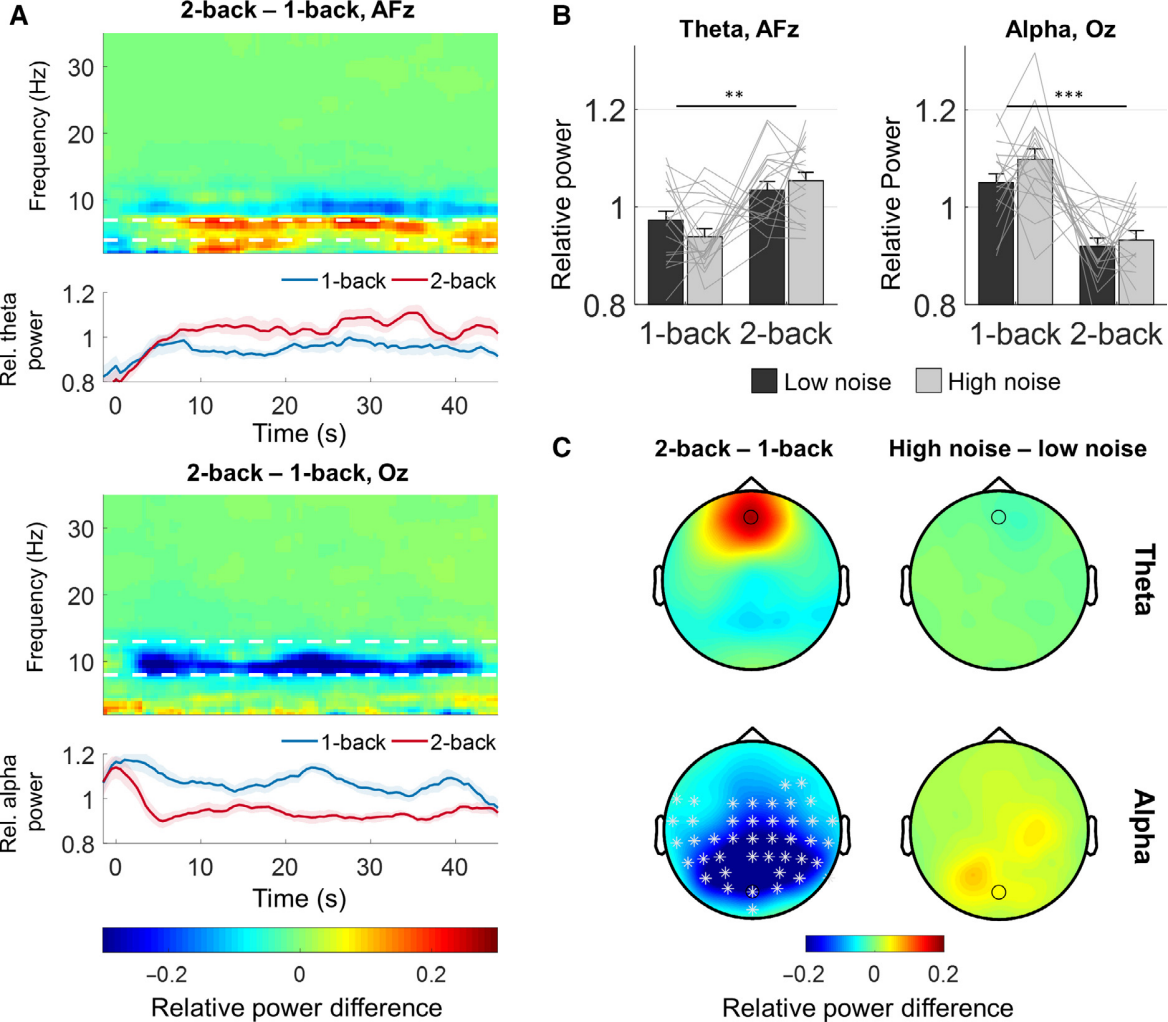
Changes in oscillatory power during the n‐back speech task. (A) Time–frequency representations (TFRs) of the power changes between the 2‐back and 1‐back tasks at frontal electrode AFz (above) and posterior electrode Oz (below). White stippled lines mark the location of the theta (above) and alpha (below) bands. Traces below the TFRs show the normalized theta band power and alpha band power in the two n‐back tasks. Shaded areas in the traces represent ± 1 SEM across subjects for each 5 s time window. (B) Trial‐mean (5–45 s) power in frontal theta (left) and posterior alpha (right). (C) Topographies showing the trial‐mean differences in theta (above) and alpha (below) power between the 2‐back and 1‐back tasks (left) and between high and low noise levels (right). Circles indicate the position of electrodes AFz and Oz. White asterisks indicate electrodes showing significant power differences between the n‐back conditions revealed by the cluster analysis (*P* < 0.01). Error bars represent ± 1 SEM **P <* *0.01, ***P < 0.001.

We first investigated changes in posterior alpha power and frontal theta power previously associated with WM load. As illustrated in Fig. 3, increasing WM load in the more difficult 2‐back task compared to the 1‐back task was associated with a decrease in posterior alpha power. An ANOVA on trial‐averaged alpha power at electrode Oz revealed a main effect of n‐back level (*F*_1,18_ = 30.15, *P *<* *0.001). Cluster‐based permutation analysis revealed a widespread cluster of posterior and central electrodes showing a significant decrease in alpha power with n‐back level (Fig. 3). Frontal theta power increased at the start of the trial and increased additionally during the 2‐back task compared to the 1‐back task (main effect at electrode Afz: *F*_1,18_ = 10.88, *P *=* *0.004). Examining the trial‐averaged theta power across all electrode sites revealed no significant clusters. No significant effects of the background noise level were observed on either alpha (*F*_1,18_ = 1.90, *P *=* *0.18) or theta (*F*_1,18_ = 0.29, *P *=* *0.60) power changes.

### Influence of WM load on speech envelope entrainment

**Figure 4:**
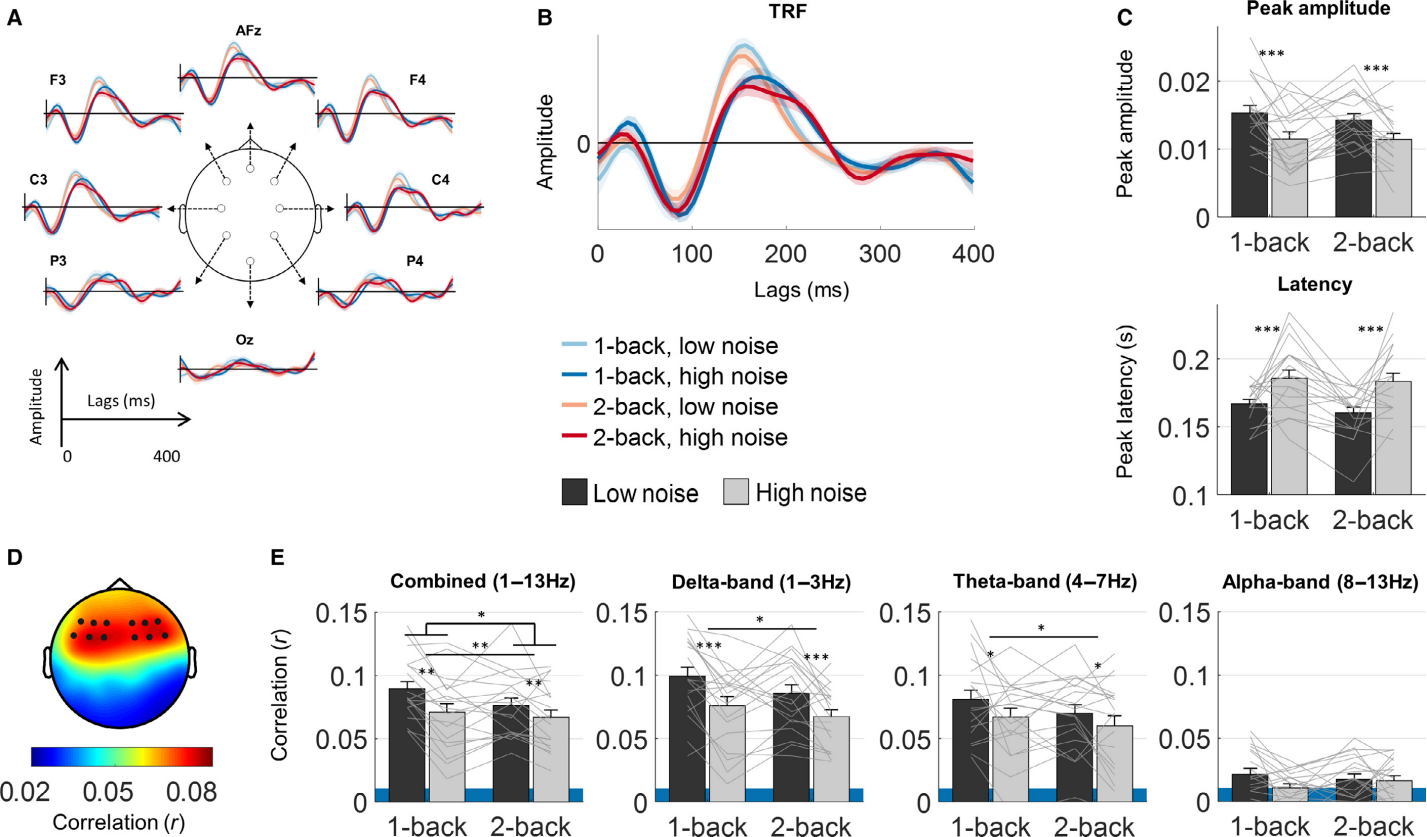
Electroencephalogram (EEG) responses to speech envelopes in the different working memory (WM) load conditions. Above (A–C): Temporal response functions (TRFs) derived from linear regression between EEG data and the speech stimulus. Below (D, E): Speech entrainment measured as the correlation between the cortical response predicted by the speech envelope and the EEG. (A) TRFs at selected electrode locations to illustrate the responses at different scalp positions. (B) TRFs averaged over frontocentral electrodes in the different experimental WM conditions. (C) The amplitude (above) and latency (below) of the late positive peak in the average TRF around 170 ms. (D) Topographical distribution of the EEG prediction accuracies (Pearson's *r*) averaged across conditions. The dots indicate the positions of the analysed frontocentral electrodes. (E) Average prediction accuracies in different frequency bands. The shaded areas represent chance‐level prediction. Error bars represent ± 1 SEM *P <* *0.05, **P <* *0.01, ***P <* *0.001.

We derived temporal response functions (TRFs, Fig. 4A and B) to analyse how low‐frequency cortical activity entrained to fluctuations in the speech envelope. The TRF can be viewed as a speech‐evoked response generalized to continuous stimuli (Lalor *et al*., 2009). In all conditions, we observed a late (~ 170 ms) positive peak in the TRF amplitudes (Fig. 4A–C). Both the amplitude and latency of the late peak were found to be affected by the background noise level (Fig. 4C). For the higher noise level, the peak latency increased (*F*_1,18_ = 20.43, *P *<* *0.001) and the peak amplitude decreased (*F*_1,18_ = 12.95, *P *=* *0.002). No significant changes in peak amplitude (*F*_1,18_ = 0.80, *P *=* *0.381) or latency (*F*_1,18_ = 0.84, *P *=* *0.371) were found for the change in n‐back level.

To quantify how precisely the cortical activity entrained to the speech envelope in the different WM conditions, we computed the correlation coefficient (Pearson's *r*) between the responses predicted by the TRF models and the measured EEG (Fig. 4D and E). The TRF models were first used to predict the low‐frequency (1–13 Hz) EEG response at 12 frontotemporal electrodes from the speech envelopes. As shown in Fig. 4D, the average prediction correlation across experimental conditions was high over frontotemporal electrodes, in accordance with previous TRF studies (Crosse & Lalor, 2014; Di Liberto *et al*., 2015). Analysis of prediction correlations between WM conditions revealed a significant interaction between n‐back level and noise level (*F*_1,18_ = 6.02, *P *=* *0.025) (Fig. 4E). The prediction values were found to decrease with increasing n‐back level (main effect: *F*_1,18_ = 10.68, *P *<* *0.005) and increasing noise level (main effect: *F*_1,18_ = 10.54, *P *=* *0.005), but the effect of the background noise was found to be larger in the 1‐back condition than in the 2‐back condition.

As previous work has pointed to different functional roles for delta‐ and theta‐band entrainment in speech coding (Ding & Simon, 2014), we also investigated the effects of behavioural WM load on speech entrainment separately in different frequency bands (Fig. 4E). This was performed by computing the prediction accuracies of TRF models estimated from EEG responses bandpass filtered in the delta, theta and alpha frequency bands. The prediction correlations were only above the noise floor in the delta and theta band, but not in the alpha band. As in the analysis of the broadband signal (1–13 Hz), the speech‐entrained response in the delta and theta bands was significantly reduced with increases in both types of WM load (Fig. 4E). Increasing the background noise level reduced prediction correlations in the delta band (main effect: *F*_1,18_ = 16.75, *P *<* *0.001) and in the theta band (main effect: *F*_1,18_ = 4.95, *P *=* *0.039). Increased WM load in the n‐back task also decreased entrainment in both the theta band (main effect: *F*_1,18_ = 7.10, *P *=* *0.016) and the delta band (main effect: *F*_1,18_ = 5.91, *P *=* *0.026).

In our analysis, we focused on entrainment between the envelope of the speech signal and cortical activity. Reduced entrainment with increased background noise levels could potentially reflect cortical entrainment to the presented noisy speech stimulus rather than the underlying speech signal. To investigate whether the cortical activity tracks the actual noisy stimulus envelope rather than the underlying speech envelope, we performed the same TRF analysis but for the envelopes of the noisy speech mixture. Prediction accuracies based on the noisy speech envelopes were significantly lower than for the envelope of the clean signals (paired *t*‐test, *t *=* *4.93, *P *<* *0.001), suggesting that the cortical activity mainly entrains to the clean speech signal rather than to the noisy sound mixture.

## Discussion

We devised an auditory n‐back task embedded in continuous speech to investigate interactions between WM load and speech processing. Consistent with previous visual n‐back paradigms (Gevins *et al*., 1997; Gevins & Smith, 2000; Pesonen *et al*., 2007; Haegens *et al*., 2014; Scharinger *et al*., 2015, 2017), increasing load with higher n‐back levels was associated with increased frontal theta band power and decreased posterior alpha power. At the same time, cortical entrainment to the speech envelope decreased with increasing WM load. Both increased background noise levels and higher n‐back levels decreased speech‐entrained responses in the delta and theta bands.

### Dynamics of alpha and theta power and pupil dilations during WM load

The continuous speech paradigm allowed us to observe the dynamics of load‐related measures over prolonged periods of WM load. Load‐specific changes in behavioural performance, EEG band power and pupil size each exhibited different dynamics over the trial duration. The observation of task‐evoked pupil dilations in the initial 5–10 s of the trial (Fig. 2C) is consistent with numerous previous pupil studies of WM load or cognitive effort in paradigms with shorter trials (Beatty, 1982; Zekveld *et al*., 2010; Koelewijn *et al*., 2012; Scharinger *et al*., 2015; Wendt *et al*., 2016). However, we also observed that this was followed by a similar decrease in pupil sizes for the remaining duration of the trial. During this decrease, the pupil dilations remained sensitive to n‐back load (Fig. 2C). Behavioural performance, on the other hand, decreased during the trial but only during the difficult 2‐back task (Fig. 2A). This could indicate fatigue. However, a similar pattern specific to the high‐load condition was not reflected in either the EEG band power or the pupil responses. In the EEG theta or alpha power (Fig. 3A), we did not find similar patterns of change throughout the trial but the individual power traces had considerable local variation.

An initial increase in theta power was observed in the beginning of the trials (Fig. 3A). This could reflect the increase of items held in WM when participants were presented with the first numbers of the sequence, consistent with visual WM tasks (Raghavachari *et al*., 2001). In the remaining parts of the trial, theta power remained high and increased additionally during 2‐back task compared to the 1‐back task. Scharinger *et al*. (2017) recently reported a similar increase in frontal theta emerging in the course of a visual n‐back task but did not observe a similar theta pattern during memorization in WM span tasks. This could suggest that theta is more specifically related to the organization and continuous update of WM items, and less to memory storage of those items. Specifically, our results are consistent with a functional role of theta oscillations for organizing multiple items in a sequential order in short‐term memory (Raghavachari *et al*., 2001; Lisman & Jensen, 2013).

Decreased alpha power for higher n‐back levels throughout the trial is also consistent with previous n‐back studies (Gevins & Smith, 2000; Pesonen *et al*., 2007; Scharinger *et al*., 2015, 2017). Reduced alpha power, however, has also been observed in a number of other complex WM tasks and may reflect the complex nature of the n‐back task. The n‐back task requires subjects to simultaneously update WM information and match stored items with the current input (Watter *et al*., 2001). A task‐related decrease in alpha power has been proposed to reflect the fact that a number of WM processes are simultaneously required for task performance (Klimesch, 1999; Scharinger *et al*., 2017). Simultaneous involvement of different WM functions in different task strategies may also explain the fact that we observed a considerable variability in alpha patterns between subjects in our data (see Fig. 3B). While some subjects may be able to search WM content before a new number is presented, others may try to match stored items each time a new speech item is heard (Watter *et al*., 2001). Different processing strategies that put different demands on the matching subtasks could potentially generate variability in the observed alpha patterns.

### Do WM processes influence speech entrainment?

Speech envelope entrainment was found to decrease with an increase in the two types of WM load examined. In visual n‐back tasks, the amplitude of P300 evoked potentials has consistently been found to be attenuated by increasing WM load at higher n‐back levels (Gevins *et al*., 1996; McEvoy *et al*., 1998; Watter *et al*., 2001; Wintink *et al*., 2001; Scharinger *et al*., 2015). This reduction has been interpreted in terms of a re‐distribution of resources between WM processes at higher load levels. Yet, decreased speech entrainment with increasing WM load, as observed in the current study, points to an interaction between WM processing and auditory processing of the speech stimulus. Thus, decreased entrainment with higher load levels may reflect a re‐allocation of WM resources at the expense of parsing of the speech stimulus.

A possible explanation for the WM‐specific reduction in speech entrainment could be an interaction between WM processing and attention (Gazzaley & Nobre, 2012). Numerous studies have demonstrated that selective attention to a particular talker reduces entrainment to ignored speech streams (Ding & Simon, 2012; Power *et al*., 2012; Zion Golumbic *et al*., 2013; O'Sullivan *et al*., 2014; Fuglsang *et al*., 2017). Auditory entrainment to speech has also been reported even in the absence of overt auditory input, for example during imagined speech (Deng *et al*., 2010; Martin *et al*., 2014). This raises the possibility that attention‐driven speech entrainment can operate entirely on internal speech representations. In a continuous updating task such as our current speech n‐back paradigm, WM processing may direct attentional focus towards the internal rehearsal of verbal items in the phonological loop. In this case, new items in the continuous speech stream compete for selective attention with verbal information currently in the phonological loop. Increasing attention towards the phonological loop for higher n‐back levels would then explain a decrease in cortical activity entrained to the ongoing speech stimulus. Such a mechanism would need to be examined more closely, for example by comparing entrainment to matching vs. mismatching search targets. We note that the observed reduction in speech entrainment during WM load is relatively small compared to the reduction in entrainment typically reported for ignored speech streams in selective attention tasks.

While higher n‐back levels reduced delta–theta entrainment, this was not accompanied by a significant reduction in TRF amplitudes. Increasing background noise levels, on the other hand, significantly attenuated and shifted the latency of the TRF peak. Consistent with this, increasing levels of continuous background noise have previously been found to increase event‐related potential latencies of both N100 and P300 components in a syllable discrimination task (Whiting *et al*., 1998). Latency shifts and attenuated amplitudes of TRFs with increasing background noise levels have also been reported previously, but only for earlier TRF components (~ 50 ms) observed in MEG component space (Ding & Simon, 2013). Our current TRF method did not reveal any clear early components, and the later peak may reflect a compound effect of early and later auditory processing.

Higher WM load levels decreased speech entrainment (Fig. 4) and, at the same time, induced load‐specific alpha–theta power changes (Fig. 3). The phase of auditory cortical activity entrained to speech has previously been suggested to be functionally coupled with alpha, theta and beta power in frontoparietal regions (Park *et al*., 2015; Keitel *et al*., 2017), but the functional significance of these couplings has not been clarified. In line with the present results, Keitel *et al*. (2017) found that reduced entrainment in the delta band was associated with increases in parietal theta power. The authors proposed that this could reflect WM involvement to compensate for weaker entrainment. Our results suggest instead that WM load, here induced by the behavioural task, reduces the speech‐entrained response. The WM‐specific power changes found in the current study (Fig. 3) also point to executive functions that are not specific to speech. However, the functional coupling involved in WM‐specific modulation of speech entrainment may be different from those observed in paradigms without specific WM tasks.

In our study, speech entrainment was defined in terms of a linear mapping between the speech envelope and the EEG signal. A decreased prediction accuracy for increasing WM load indicates that WM load influences how accurately cortical activity tracks acoustic amplitude variations in the speech signal. Such a picture is consistent with the notion of a general oscillatory network that modulates activity in sensory cortices in a top‐down manner (Schroeder & Lakatos, 2009). While conceivable, this conclusion may be premature based on the current results in isolation. Delta–theta envelope entrainment has also been reported for nonspeech signals or unintelligible speech sounds (Lalor *et al*., 2009; O'Sullivan *et al*., 2014; Millman *et al*., 2015). In speech signals, however, the amplitude envelope correlates with the quasi‐rhythmic variations in higher‐level speech features, such as the onsets of phonemes or syllables. Cortical entrainment in speech processing has also been suggested to be related to parsing of such high‐level speech units (Ghitza, 2011; Giraud & Poeppel, 2012; Di Liberto *et al*., 2015; Zoefel & VanRullen, 2016), and WM load could modulate speech processing at any or several different levels of speech processing.

Although we suggest that the effects of background noise on delta–theta entrainment reflect WM load, changes in entrainment could potentially have been related to the acoustic degradation of the sound envelope. To investigate whether a reduction in entrainment might reflect the fact that cortical activity entrains to the noisy signal, we compared entrainment to the clean speech signal (without noise) with entrainment to the noisy sound stimulus actually presented to the listeners. In agreement with previous results (Ding & Simon, 2013; Fuglsang *et al*., 2017), this suggested that cortical activity predominantly entrained to the underlying speech signal rather than to the noisy sound mixture. While this suggests an effect of WM load induced by the noise interference, our design does not allow us to completely dissociate the effects of acoustic degradation of the sound signal from inhibitory load caused by these degradations. Alternative paradigms that burden WM inhibitory load without affecting the acoustic stimulus, for example by presenting incongruent speech features, might further dissociate these effects.

### Limitations

In our study, we used long continuous speech stimuli (~ 45–55 s) to investigate auditory entrainment during WM load. However, simultaneously examining WM‐dependent effects on speech entrainment and on oscillatory power involves a trade‐off in terms of experimental design. TRF methods are generally found to be more robust to EEG artefacts but they require long trials for estimating the stimulus‐response mapping at lower frequencies. Although the TRF methods allow neural responses to continuous speech to be examined, longer trials are not optimally suited to track spectral power changes in the EEG. Power estimates are more susceptible to EEG artefacts and activity unrelated to the stimulus or task. In the current study, we observed a substantial individual variability in the considered power measures. It is possible that alternative paradigms using shorter trials and more trial averages would be more sensitive to the oscillatory power changes associated with these WM tasks and could reveal additional effects. Also, the current analyses relied on EEG power estimates in fixed frequency bands, although the spectral characteristics of alpha and theta power may vary considerably between subjects (Haegens *et al*., 2014). The group analyses of theta–alpha power may thus be susceptible to between‐band leakages.

## Conflict of interests

The authors declare no conflict of interests.

## Author contributions

J.H. conceived the study; J.M., S.F. and J.H. designed the experiment; J.M collected the data; J.M., S.F. and J.H. analysed the data and interpreted the results; and all authors wrote the paper.

**Abbreviations**
CDF: cumulative distribution function
EEG: electroencephalogram
EOG: electrooculogram
ERP: event‐related potential
MEG: magnetoencephalogram
SNR: signal‐to‐noise ratio
TFR: time–frequency representation
TRF: temporal response function
WM: working memory

## Data Availability

The EEG and audio data are available at zenodo.org: https://doi.org/10.5281/zenodo.1158410.
