# Supplementary material for: Cortical oscillations and entrainment in speech processing during working memory load
Source: Eur J Neurosci. 2018 Feb 16;51(5):1279–89. doi: 10.1111/ejn.13855 (PMC7155003; doi:10.1111/ejn.13855)
Supplement: Supplementary file 1 [file EJN-51-1279-s001.pdf]

EJN European Journal of Neuroscience FENS Federation of European Neuroscience Societies

Cortical oscillations and entrainment in speech processing during working memory load

Jens Hjortkjær, Jonatan Märcher-Rørsted, Søren A. Fuglsang & Torsten Dau

Review timeline:

Submission date:	23 June 2017
Editorial Decision:	14 August 2017
Revision received:	22 November 2017
Editorial Decision:	19 December 2017
Revision received:	25 January 2018
Accepted:	26 January 2018

Editor: John Foxe

1st Editorial Decision 14 August 2017

Dear Dr. Hjortkjær,

Your manuscript has now been reviewed by two external reviewers and ourselves. The reviews collectively indicate that your experiments generated new and important information. However, there are several substantial issues that need to be clarified and resolved before we can consider your manuscript further for publication in EJN.

As you will read in the provided reviews below, there are strong suggestions to expand your analyses to encompass a more complete interrogation of the entire frequency band and for you to add better illustrations of the topographies of the effects that you report. It would provide much greater confidence in the results you report if the reader can see that the effects are consistent across a physiologically plausible grouping of electrodes (rather than at a single scalp site). Of course, it is appreciated that the primary hypothesis-driven analysis was concentrated on specific bands and that any such additional analyses would need to be couched as exploratory. In a similar vein, reviewer #2 points to inconsistencies in the way that analyses have been applied to different aspects of your data matrix. Please also provide a more complete and detailed description of your data analysis process, including how ICA components were removed and how normalization was conducted. There are also suggestions for additional literature that should be considered to improve scholarship. You will note that we all have concerns about the number of participants that survive to full analysis and feel that it will be appropriate for you to acknowledge potential issues of power. Better still, of course, would be to add new data to improve the power of the study.

Please also attend to the following issues:
1) You referencing is not in EJN style – all authors of cited papers should be included in the reference list
2) A data sharing statement needs to be explicitly included in the manuscript itself, and we strongly encourage you to provide as much of the data as possible on a publicly available repository (e.g. Figshare)
3) Please do not embed your figures in the text in the next version
4) Replace your barcharts with scatterplots or some other more informative hybrid depictions - see http://onlinelibrary.wiley.com/doi/10.1111/ejn.13400/epdf

If you are able to respond fully to the points raised, we would be pleased to receive a revision of your paper within 12 weeks.

Thank you for submitting your work to EJN.

Kind regards,

John Foxe & Paul Bolam
co-Editors in Chief, EJN

EJN European Journal of Neuroscience FENS Federation of European Neuroscience Societies

Reviews:

Reviewer: 1 (Til Ole Bergmann, University of Tuebingen, Germany)

Comments to the Author
Hjortkaer et al. investigated the impact of working memory (WM) load in speech processing, and in particular on (i) the WM dependent modulation of alpha and theta power and (ii) on the entrainment of cortical activity to the attended speech envelope. They designed an auditory n-back task with the factors n-back level (1- or 2-back) and noise level (low and high background noise) that tested the 'updating' and the 'inhibition' aspect of WM, respectively, and recorded 64-channel EEG and pupil dilation during the task. The authors showed that theta power increases with WM updating demands (n-back level), whereas alpha power increases with inhibition demands (noise level). Moreover, speech entrainment of cortical activity was observed in the theta and delta bands, while only the theta band entrainment was modulated my WM load. The paper is well written and the study appears to be carefully designed, controlled, and executed. The methods are sound and the interpretation of the data is backed up by the respective statistics. While the number of subjects (N = 12 in the final analysis) is on the lower bound, the study results are consistent and the statistical significances convincing. The study targets the relevant issue of how speech related neuronal entrainment is modulated by working memory demands, a crucial component in speech processing. I have only a few specific comments the authors should address before publication of the manuscript.

1. It remains unclear how selective the power results are for the theta and alpha band, since those bands were extracted (squared absolute values of band-pass filtered data) and analysed based on a priory hypothesis. However, it would be helpful, to additionally provide results for a larger frequency spectrum to assess the specificity in the frequency domain. I suggest to use a moving window FFT (or the method applied so far also for all the other frequency bins) to additionally evaluate a larger frequency range (e.g., 1 to 45 Hz) and show whether and how other frequencies were modulated as well. Thus Fig. 3 A and D could be complemented by a time-frequency representation (TFR).

2. Similar to the above, the analysis of the entrainment is based on preselected frontal and fronto-central EEG channels. Again I would suggest to show a topographical representation (topoplots like in Fig. 3C) of the entrainment effect at all 64 channels, either just for illustration purposes or using the cluster based permutation statistics implemented in FieldTrip to control for multiple comparisons. In any case, it would be helpful to see how this effect distributes.

3. Regarding the decline of behavioural performance across a 2-back task trial: Maybe a correlation with pupil dilation, theta or alpha power would become visible, when explicitly testing the correlation of those variables with behavioural performance, extracting their values e.g., for the 4 different time windows used in Fig. 2A?

4. Regarding the ICA artefact rejection procedure: It would be good to state what kind of components were removed, how many of each kind, and based on which criteria those components were selected.

5. P11 "Theta power was defined relative to the IAF as the frequency range from IAF/2 to IAF/2+2 Hz." Why? Please explain and provide reference for this procedure.

6. P11: "[…] the power measures for each of the four conditions were normalized by the individual average band power across all trials for each subject." Please specify the normalization (ratio? Z-transform?).

Reviewer: 2 (Eline Borch Petersen, Eriksholm Research Centre, Denmark)

Comments to the Author
The goal of this paper is to establish the effects of varying both the storage (n-back) and processing (noise level) aspects of the WM function in an auditory processing task. More specifically, the effects are investigated for pupilometry data, cortical activity, as well as on the speech-envelope entrainment of different oscillatory bands.
While the paper addresses the individual roles of the different WM functions (inhibition and updating) which is of interest to the scientific community, major revisions are needed to unify the analysis methods and refine some aspects of the results and conclusions consequently drawn from it.

Besides the more specific concerns (to be found in the attached file), listed below are some major concerns that should be addresses to make the paper suitable for publications.

1. The paper aims at testing the inference between WM processing and speech entrainment, however the comparison seems inconsistent. Alpha and theta power has previously been linked to WM processing, whereas the entrainment of delta and theta activity to the speech envelope has not yet been clearly linked

to WM processing as detailed as tried in the current paper. The comparison between cortical oscillatory activity and speech entrainment is especially inconsistent in the methods used for deriving the two measures: Whereas the power (alpha and theta) is calculated based on individualized measures of IAF, the speech entrainment is derived as a general measure of activity within a band of non-individualized (general) frequencies. This makes it very difficult to figure out whether the differences/likelihoods between power and entrainment are actually valid or resulting from applied method.
Using the IAF to calculate the alpha and theta range is a recognized, although not often used, approach. However, the approach should in my opinion be used for both the power and the entrainment investigations. The average individual IAF of 9.83+/- 0.61 Hz result in some subjects having IAFs as low as 8.6 Hz (9.8-2*0.6, mean minus 2 standard deviations), result in alpha frequency bands starting at frequencies between 6.6 to 11 Hz between individuals and extending 4 Hz. This can by no means be compared to the 8-15 Hz alpha band used for the speech entrainment results. Just the sheer difference in the range of the two alpha bands (4 Hz for the individual alpha and 7 Hz for the general) raise some concern. It is suggested to use one, either the general or the individualized, approach for calculating the frequency ranges.

2. The introduction is generally missing literature describing the clear link between the WM processing and speech entrainment. Although three possible scenarios linking the two concepts are provided (section 2 of the introduction), only one is based on studies actually involving speech entrainment. It would be nice with some background thoughts/literature describing the link in more details. Potentially, a more detailed description of the findings of Park et al and Keitel et al would be sufficient.

3. For the analysis of the power (figure 3), the conclusions are based on effects found in just a single electrodes (Oz or Fz, corresponding to 1.6% of the data collected across the 64 electrodes). To strengthen the conclusion, the effects should be consistent across a larger topographic area (frontal or parietal). It is suggested to perform an analysis based on an average of several electrodes or, preferably, do a cluster-based analysis to identify relevant clusters of electrodes while at the same time adjusting for the multiple-comparisons problem.

4. Considering that the paper presents results from 11 listeners, with a relatively high amount of the data being removed due to noise, and with some of the results having p-values very close to 0.05, I'm wondering whether there are any statistical concerns. It would suit the paper to comment this.

5. Although the speech material is developed to mimic natural speech, it does not seem right to describe the speech material as 'natural continuous speech'. A more appropriate term might be 'synthesize/simulated/mimicked natural speech'.

6. Throughout the manuscript it is advised that the authors use more adjectives to describe words such as 'resources' and 'inhibition' to clarify whether the words refer to general cognitive, working memory, or neural mechanisms. Also clear definitions of the terms WM demands, WM functions, and WM load would be nice.

Authors' Response 22 November 2017

Below we list our responses (in black) to each of the comments in the reviews (in blue). References to the revised manuscript are indicated by P (page) and L (line) numbers. In the revised manuscript, changes are marked in yellow.

Editors

As you will read in the provided reviews below, there are strong suggestions to expand your analyses to encompass a more complete interrogation of the entire frequency band and for you to add better illustrations of the topographies of the effects that you report. It would provide much greater confidence in the results you report if the reader can see that the effects are consistent across a physiologically plausible grouping of electrodes (rather than at a single scalp site). Of course, it is appreciated that the primary hypothesis-driven analysis was concentrated on specific bands and that any such additional analyses would need to be couched as exploratory. In a similar vein, reviewer #2 points to inconsistencies in the way that analyses have been applied to different aspects of your data matrix. Please also provide a more complete and detailed description of your data analysis process, including how ICA components were removed and how normalization was conducted. There are also suggestions for additional literature that should be considered to improve scholarship. You will note that we all have concerns about the number of participants that survive to full analysis and feel that it will be appropriate for you to acknowledge potential issues of power. Better still, of course, would be to add new data to improve the power of the study.

We would like to thank the reviewers for their constructive criticism and comments. To meet the general concern about the low number of participants, we have acquired additional EEG data from additional 7

subjects (now N=22) to increase the statistical power. The revised manuscript is based on new analyses of the data following the suggestions of the reviewers. We now include illustrations of the power changes over a larger frequency range. In addition to hypothesis-driven tests, we have included cluster-based tests over electrodes, as discussed in more detail in our responses below. The reviewers also asked for a more consistent definition of the frequency bands between the spectral power analysis (previously relying on individual alpha and theta bands) and the entrainment analysis (in fixed frequency bands). We have now changed the analysis to rely on the same fixed bands in the two types of analysis. Non-individualized frequency bands introduce the problem of leakages between the neighboring theta and alpha bands in the group average results, but we agree with the reviewers that it makes the results more readily comparable. We now define non-overlapping frequency bands delta (1-3 Hz), theta (4-7 Hz) and alpha (8-13 Hz) to minimize between-band leakages. Overall, the results are similar to the ones initially reported, with a few exceptions. The effect of noise level on alpha band power is no longer statistically significant. We have therefore removed all discussion related the effects of noise on alpha power. In the entrainment analysis (based on the new frequency band definitions), we again find that WM load decreases entrainment, and find the effect in both the delta and theta bands. The decreased amplitude of the late TRF peak in the 2-back task is no longer statistically significant. Although we have acquired more data, we acknowledge that our experimental design with long speech stimuli may not be optimal for detecting spectral power changes and that faster designs could be more sensitive for examining these aspects. We now discuss this in a 'limitations' section of the revised paper.

Please also attend to the following issues:

1) You referencing is not in EJN style – all authors of cited papers should be included in the reference list

We have corrected the referencing.

2) A data sharing statement needs to be explicitly included in the manuscript itself, and we strongly encourage you to provide as much of the data as possible on a publicly available repository (e.g. Figshare)

We would be happy to share the data. We will make the EEG and audio data publicly available at zenodo.org. A data sharing statement has been included (P21, L618-619). The Matlab code used for analyzing the EEG response functions is publicly available and a reference has been included in the revised manuscript (P11, L328-329).

3) Please do not embed your figures in the text in the next version

The figures have now been removed from the text.

4) Replace your barcharts with scatterplots or some other more informative hybrid depictions - see http://onlinelibrary.wiley.com/doi/10.1111/ejn.13400/epdf

We have revised all figures based on these recommendations and additional comments. Bar charts and other plots now additionally display the data for each individual subject.

Reviewer #1

Comments to the Author

Hjortkaer et al. investigated the impact of working memory (WM) load in speech processing, and in particular on (i) the WM dependent modulation of alpha and theta power and (ii) on the entrainment of cortical activity to the attended speech envelope. They designed an auditory n-back task with the factors n-back level (1- or 2-back) and noise level (low and high background noise) that tested the 'updating' and the 'inhibition' aspect of WM, respectively, and recorded 64-channel EEG and pupil dilation during the task. The authors showed that theta power increases with WM updating demands (n-back level), whereas alpha power increases with inhibition demands (noise level). Moreover, speech entrainment of cortical activity was observed in the theta and delta bands, while only the theta band entrainment was modulated my WM load. The paper is well written and the study appears to be carefully designed, controlled, and executed. The methods are sound and the interpretation of the data is backed up by the respective statistics. While the number of subjects (N = 12 in the final analysis) is on the lower bound, the study results are consistent and

the statistical significances convincing. The study targets the relevant issue of how speech related neuronal entrainment is modulated by working memory demands, a crucial component in speech processing. I have only a few specific comments the authors should address before publication of the manuscript.

1. It remains unclear how selective the power results are for the theta and alpha band, since those bands were extracted (squared absolute values of band-pass filtered data) and analysed based on a priory hypothesis. However, it would be helpful, to additionally provide results for a larger frequency spectrum to assess the specificity in the frequency domain. I suggest to use a moving window FFT (or the method applied so far also for all the other frequency bins) to additionally evaluate a larger frequency range (e.g., 1 to 45 Hz) and show whether and how other frequencies were modulated as well. Thus Fig. 3 A and D could be complemented by a time-frequency representation (TFR).

We thank the reviewer for this comment. As suggested, we have computed the time-frequency representations across the larger frequency range. The time-frequency representations are now shown in the updated Figure 3A.

2. Similar to the above, the analysis of the entrainment is based on preselected frontal and fronto-central EEG channels. Again I would suggest to show a topographical representation (topoplots like in Fig. 3C) of the entrainment effect at all 64 channels, either just for illustration purposes or using the cluster based permutation statistics implemented in FieldTrip to control for multiple comparisons. In any case, it would be helpful to see how this effect distributes.

We have added a topographical map of the entrainment effect to Figure 4D for illustration. The entrained speech response is seen at fronto-central electrodes, highly similar to topographical distributions reported in previous TRF speech studies (e.g. Di Liberto et al. 2015). We do not expect to find WM modulation of entrainment at sites where there is no speech entrained response in the first place, and maps of differences in entrainment over all electrodes (and statistics on this) may be confusing or misleading.

3. Regarding the decline of behavioural performance across a 2-back task trial: Maybe a correlation with pupil dilation, theta or alpha power would become visible, when explicitly testing the correlation of those variables with behavioural performance, extracting their values e.g., for the 4 different time windows used in Fig. 2A?

Although a correlation with behavioral performance over time would be highly interesting, we are concerned that correlation values between only 4 data points could be misleading. The EEG theta power trace displays an initial rise during the first ~10 sec of the trials. This could be related to WM processing but could also represent an unknown physiological effect. Following the reviewer's comment, we have examined the correlation between the EEG trace in alpha and theta power and the pupil dilations, but this did not reveal any significant relationship. It is still possible that a more thorough analysis of the relation between the EEG and pupil data could be interesting, but we think that this would be outside the scope the current paper.

4. Regarding the ICA artefact rejection procedure: It would be good to state what kind of components were removed, how many of each kind, and based on which criteria those components were selected.

We have now inserted this information in the paper (P10, L287-292). ICA components were rejected based on three categories of artefactual content: EOG artefacts, muscle artefacts, and cardiac-related artefacts. On average, 4.2 +/- 1.7 components were rejected from each subject (range 2-7), comparable with previous literature. Of the rejected components, 2.4+/- 1.3 components were attributed to EOG artefacts, as they were highly correlated with the EOG electrodes with strong weights at frontal scalp regions. The remaining 1.8 +/- 1.4 components were attributed to either muscle or cardiac-related artefacts.

5. P11 "Theta power was defined relative to the IAF as the frequency range from IAF/2 to IAF/2+2 Hz." Why? Please explain and provide reference for this procedure.

Concerns regarding the definition and use of IAF were also raised by Reviewer 3. We have now changed the analysis to rely on fixed frequency bands instead.

6. P11: "[...] the power measures for each of the four conditions were normalized by the individual average band power across all trials for each subject." Please specify the normalization (ratio? Z-transform?).

The power measure was normalized per subject by taking the ratio of the band power for a given condition and the global average power in that band. The normalization is now described in more detail on P10-11, L307-313.

Reviewer #2

Comments to the Author
The goal of this paper is to establish the effects of varying both the storage (n-back) and processing (noise level) aspects of the WM function in an auditory processing task. More specifically, the effects are investigated for pupilometry data, cortical activity, as well as on the speech-envelope entrainment of different oscillatory bands.
While the paper addresses the individual roles of the different WM functions (inhibition and updating) which is of interest to the scientific community, major revisions are needed to unify the analysis methods and refine some aspects of the results and conclusions consequently drawn from it.

Besides the more specific concerns (to be found in the attached file), listed below are some major concerns that should be addresses to make the paper suitable for publications.

1. The paper aims at testing the inference between WM processing and speech entrainment, however the comparison seems inconsistent. Alpha and theta power has previously been linked to WM processing, whereas the entrainment of delta and theta activity to the speech envelope has not yet been clearly linked to WM processing as detailed as tried in the current paper. The comparison between cortical oscillatory activity and speech entrainment is especially inconsistent in the methods used for deriving the two measures: Whereas the power (alpha and theta) is calculated based on individualized measures of IAF, the speech entrainment is derived as a general measure of activity within a band of non-individualized (general) frequencies. This makes it very difficult to figure out whether the differences/likelihoods between power and entrainment are actually valid or resulting from applied method.
Using the IAF to calculate the alpha and theta range is a recognized, although not often used, approach. However, the approach should in my opinion be used for both the power and the entrainment investigations. The average individual IAF of 9.83+/- 0.61 Hz result in some subjects having IAFs as low as 8.6 Hz (9.8-2*0.6, mean minus 2 standard deviations), result in alpha frequency bands starting at frequencies between 6.6 to 11 Hz between individuals and extending 4 Hz. This can by no means be compared to the 8-15 Hz alpha band used for the speech entrainment results. Just the sheer difference in the range of the two alpha bands (4 Hz for the individual alpha and 7 Hz for the general) raise some concern. It is suggested to use one, either the general or the individualized, approach for calculating the frequency ranges.

Thank you. We agree with the reviewer that frequency bands analyzed in terms of power associated with WM processing and in terms of speech envelope entrainment should be consistent. We have redone all analyses using the same fixed frequency bands in both types of analysis. The results of our new analyses are qualitatively similar to the initial ones, with the exception of the effect of the noise level on alpha power that is no longer statistically significant. Our initial IAF power analysis was based on previous studies of alpha power showing considerable individual differences. Although similar frequency bands are considered in the two types of analysis, we do not consider alpha or theta power related to WM processing to have the same cortical origin as the entrained (theta) activity. Yet, as the reviewer points out, a consistent definition of the frequency bands is still preferable and renders the results more comparable with past literature. We hope that our revision addresses this.

2. The introduction is generally missing literature describing the clear link between the WM processing and speech entrainment. Although three possible scenarios linking the two concepts are provided (section 2 of the introduction), only one is based on studies actually involving speech entrainment. It would be nice with some background thoughts/literature describing the link in more details. Potentially, a more detailed description of the findings of Park et al and Keitel et al would be sufficient.

We are not aware of any previous study describing or demonstrating a link between WM processing and speech entrainment. We acknowledge that thoughts about a possible relationship are speculative given the lack of previous work. We have revised the introduction, also including a separate section with more details about the findings of Park et al and Keitel et al. We hope that the motivation for studying the link between WM processing and speech entrainment has become clearer.

3. For the analysis of the power (figure 3), the conclusions are based on effects found in just a single electrodes (Oz or Fz, corresponding to 1.6% of the data collected across the 64 electrodes). To strengthen the conclusion, the effects should be consistent across a larger topographic area (frontal or parietal). It is suggested to perform an analysis based on an average of several electrodes or, preferably, do a cluster-based analysis to identify relevant clusters of electrodes while at the same time adjusting for the multiple-comparisons problem.

As suggested, we have computed the cluster effects of spectral power differences over all electrodes using permutation statistics. We computed the group-level spatial effect of trial-averaged power differences over all electrodes using *t*-tests with a cluster-defining threshold (cluster alpha) of $p<0.01$, a cluster-level threshold of $p<0.01$, and a neighborhood extent of 40 mm (as implemented in FieldTrip's ft_freqstatistics). In the revised paper, we still report the results of our initial single-channel power analysis based on an a priori hypothesis of alpha modulation at posterior channels and theta modulation at frontal channels. This hypothesis was based on previous literature (e.g., Scharinger et al. 2015) showing localized effects in theta and alpha power related to WM processing. In the new analysis, the bands over which theta and alpha power are computed are fixed across subjects (in response to the comments above). Since the alpha band in particular varies across individuals, an exploratory analysis over all electrodes is subject to the shortcoming that possible leakages between the alpha and theta bands in the group data.

4. Considering that the paper presents results from 11 listeners, with a relatively high amount of the data being removed due to noise, and with some of the results having p-values very close to 0.05, I'm wondering whether there are any statistical concerns. It would suit the paper to comment this.

This point is well taken. We have acquired EEG data from an additional 7 subjects, so the total number of subjects is now 22. The spectral power analysis is now based on 19 subjects (3 of the original subjects were excluded, none of the new subjects were excluded). We do not think that the reported effects in the revised paper can be considered close to our statistical threshold. In the revised discussion, we have inserted a 'limitations' section to acknowledge potential limited sensitivity for detecting spectral power changes in our experimental design. We used long trials to investigate continuous speech processing with TRF methods. This also means that we had a limited amount of trial averages, compared to WM studies using faster paradigms. While TRF methods are robust to noise, the power estimates are generally more vulnerable to EEG artefacts. For this, we found it best to take a conservative approach to EEG noise removal. We acknowledge that a faster paradigm with more trial averages could be more sensitive to WM-dependent power changes, and could potentially reveal additional effects not seen in the current study. However, the significant theta and alpha power changes we observe with n-back level are highly consistent to those reported in previous n-back studies.

5. Although the speech material is developed to mimic natural speech, it does not seem right to describe the speech material as 'natural continuous speech'. A more appropriate term might be 'synthesize/simulated/mimicked natural speech'.

Throughout the manuscript, we have either changed 'natural speech' to 'mimicked natural speech' as suggested, or removed the term.

6. Throughout the manuscript it is advised that the authors use more adjectives to describe words such as 'resources' and 'inhibition' to clarify whether the words refer to general cognitive, working memory, or neural mechanisms. Also clear definitions of the terms WM demands, WM functions, and WM load would be nice.

In the context of our current study, we define WM load operationally in terms of the n-back task (ie n-back level), and we discuss the specific WM functions that has been hypothesized to be involved in performing this task (e.g., P4, L114-121 and P17, L498-507). We hope that these definitions are clearer in the revised manuscript.

Specific comments:

The specific comments refer to specific pages (P) and lines on the page (L). Figures and legends are commented separately below.

Thank you for these many detailed comments. They have helped us improve the paper greatly.

Generally

References are missing from the reference list, e.g., Doelling et al. and Händle et al. Inconsistent whether number and unit, as well as number and equal-sign, are separated by a space or not.

Thank you. The two missing references have now been included in the revised manuscript. Inconsistencies in number-unit and number-equal sign spacing have now been corrected as: Number Unit, and Number=Answer.

Abstract

Although in the keywords, the abstract does not mention the pupilometry results. On the contrary a major part of the paper is on speech entrainment, which is not a keyword

We now mention of the pupil results in the abstract and have added speech entrainment to the list of keywords.

P2.L13 Please provide an intermediate conclusion for the result of the power analysis

We have inserted the following sentence: "The observed alpha-theta power changes are consistent with visual n-back paradigms, suggesting general oscillatory correlates of WM processing load"

P2.L15 Besides 'delta-theta' (maybe write delta and theta) you also included alpha.

We have changed this to 'low-frequency cortical activity (<13 Hz)'.

P2.L19 I feel that the final sentence concluding the study could be stronger

We have changed the final sentence of the abstract.

Introduction

P3.L4 This sentence reads like selective attention completely eliminates the neural tracking of unattended/disturbing speech, which is not the case.

We have changed this. The sentence now reads: "Selective attention is known to modulate this response by enhancing the entrainment between low-frequency cortical activity and the speech stream that the listener is attending to, relative to the ignored stream" (P3, L72-75).

P3.L12 Give the frequency interval for the theta range as well

The frequency range for theta is mentioned in the beginning of the paragraph (P3, L70).

P3.L12 Park et al looks mainly at the coupling of phase information to speech entrainment and not the relation with band power. This should be stated.

Done.

P3.L17 For the sake of following the reasoning, I recommend rearranging the three scenarios to first mention the one that is based on previous scientific findings (from Ding and Simon, increased WM load resulting in no change in the entrainment), followed by the one based on visual studies (increased WM load resulting in a decreased entrainment), and finally the 'speculative' relation (increased WM load resulting in increased entrainment)

We have reorganized this section as suggested, thank you.

P4.L10 As the functional inhibition theory linked to alpha power states that increased WM demands result in increased neural inhibition (alpha power) of task irrelevant regions and processes, it is not recommended to include results from visual WM experiments in this paragraph. Auditory WM experiment

often causes increased alpha power in the visual cortex, which innately will not be inhibited during visual WM experiment. Alpha power results in auditory and visual experiments can therefore be very contradicting, but still be explained by the same functional mechanism.

We do not think that the increase in alpha power at posterior channels that has often been reported in auditory tasks needs to reflect functional inhibition of visual cortex or visual processing specifically. On the contrary, we find, as argued in the paper, that the consistency of this alpha pattern in visual, auditory and motor tasks involving inhibition of information suggests a generic role of posterior alpha oscillations in WM tasks that is not specifically related to visual processing.

P4.L24 Is this sentence is relevant for the rest of the paragraph.

We are unsure which sentence might be irrelevant here.

P4.L25 Again, be careful with comparing findings from visual and auditory WM experiment

Please see our response to this point above.

P4.L27 Reference missing from reference list

The reference (Händel et. al 2011) has now been added to the reference list (P23, L676).

P5.L1 Please specify that the study by Pichora-Fuller show a behavioural increased in WM load

This has been specified (P4, L126).

P5.L23 Is a 1 minute trial really showing effects of prolonged WM load?

Yes, we find that 1 minute is a long stimulus duration for a WM task of this type. For comparison, previous studies that have used the same or similar WM tasks typically used much shorter stimulus trial durations, usually below 10 secs:

Chen and Huang (2016) ~3s long trials (visual, n-back)
Fockert et al (2001) 5-10s long trials (visual, n-back mixture)
Foxe and Snyder (2011) 2-4s long trials (audio-visual, cueing paradigm)
Gevins and Smith (1996) 4-5s long trials (visual-semantic, n-back)
Haegens et al (2014) 6s long trials (visual, n-back)
Händel et al (2011) 4s long trials (visual, inhibition)
Jensen et al (2002) 4s long trials (visual, Sternberg)
Jensen et al (2010) 4s long trials (visual, inhibition)
Kawasaki et al (2010) 16s long trials (audio-visual, retention/calculation task)
Obleser et al (2012) ~6.1-s long trials (auditory retention)
Wöstmann et al (2017) 6.6-s long trials (auditory retention)

We were interested in a measure of continuous speech processing and thus adapted a paradigm with longer stimuli, comparable to the durations often considered in continuous speech-entrainment studies. We now comment on this fact in the revised manuscript.

Materials and Methods

P6.L3 Please state the mean age as well as the range

This has now been included (P6, L168).

P6.L4 Where the hearing thresholds measured or self-reported?

Normal hearing was self-reported. This has now been specified on P6, L170.

P6.L12 Was the speaker male or female?

The speaker was male. We now specify this (P6, L178).

P7.L2 Which speech-reception threshold was the 100% performance estimated from?

The speech reception threshold was estimated by means of a separate psychoacoustic test where four normal-hearing subjects listened to individual speech tokens embedded in speech-shaped noise. We have now specified this (P7, L195-200).

P7.L3 As I see, you individualize the noise based on the SRT, much like Petersen et al 2017 has done before, correct? Then I would not use the term 0 dB SNR as that indicates 0 dB between noise and speech, but rather 0 dB SRT100 to follow the same notation from Petersen et al (if a notation other than high/low noise level is needed). Please state the average and standard deviation of the SRT100 across listeners. Furthermore, a description of how the low noise level was calculated is missing from the manuscript.

The noise level was not individualized since we only included normal-hearing participants. SRTs were found for four normal-hearing subjects not participating in the study. Based on these data, a global SRT100 was estimated to be 0dB SNR. The lower noise level was chosen to be 10dB higher in SNR than the empirically estimated SRT100. We have now specified this in more detail (P7, L195-200).

P7.L25 The description of Figure 1 does not comply with the trial-outline described in the text. Figure 1 shows no silent baseline. At some point in the article, please state the choice of having a silent baseline for the pupilometry, which in most studies have a noisy baseline (Wendt, 2016).

We have revised Figure 1 and specified the trial outline in more detail in the revised manuscript (P8, L225-235). Since we used two different noise levels, we used a silent baseline to be able to dissociate the effects of the noise level.

P8.L2 Please note whether the subjects were instructed to press the button with any particular finger.

No instruction was given in terms of using a particular finger for responding. This detail is now included on P8, L234.

P8.L10 On average, how many targets were there in each trial?

Lists contained either 4 (15 out of 20 lists) or 3 (5 out of 20 lists) n-back targets. This detail is now specified (P8, L. 242-243).

P8.L9 Was the level of background light controlled in any way to account for the pupil measurements? Or was it kept constant for all participants?

The light level was kept at a constant level for all participants. This information is now included on P9, L250.

P8.L12 Please state exactly when the background noise was turned on. Does changing the background noise level not affect the pupil-measurements? Is it not customary to keep the level of background noise constant and change the speech level when measuring pupilometry?

Thank you for pointing this out. It is correct that changing the background noise level can affect the pupil outcomes. However, in our results, this was not the case, as both mean pupil dilation, as well as maximum pupil dilation were unaffected by the change in noise level. Regarding the presentation of the noise, this information has now been included on P8, L230-232.

P9.L15 Were the recording-reference really the left and right mastoids? Or were they re-referenced later on? If so, please specify the recording reference.

“Reference” has now been changed to “Additional” (P9, L258). The recording reference was the CMS and DRL electrodes.

P10.L10 Please add ‘re-referenced to the averaged…’

This has now been included on P10, L278.

P10.L11 Please comment on what effect is has to use the average reference instead of the linked mastoids. It must somehow affect the topographic distribution of the alpha and theta power and maybe even the TRF response. Preferably, all subjects should have the same reference, how does it affect the results if either this subject is excluded or all data is re-referenced to the grand average?

We have computed the analysis while leaving out the subject with a noisy mastoid electrode. This did not affect the results in terms of the observed effects. We agree that the topographic distribution of power may be influenced by the choice of reference, but the average reference in a single subject did not influence the results reported here.

P10.L12 As it is written, it sounds like bad channels were removed and then noisy channels were interpolated, is this correct? Or were bad/noisy channel identified and interpolated? Please specify.

Both bad/noisy channels were identified, removed and interpolated. This has now been specified on P10, L280.

P10.L17 Please also add the Winkler reference here.

This has now been included on P10, L285, thank you.

P10.L18 As the number of independent components is reduced whenever channels are interpolated, please provide the number of rejected components in % of the total number of components.

The resulting percentage of rejected components was calculated to be 6.9%+/-2.6% .This has now been included in the manuscript on P10, L288.

P10.L22 Were 10+/-4 trials out of 40 possible trial removed? That is a very high number of trials is it not? In theory, some participants might only have a few minutes of data from one of the conditions if an average of 25% of the data is removed.

Our rejection criteria for subjects ensured that at least 50% of trials in a given condition were included in the analysis. Therefore, at least 5 minutes of data were analyzed in each condition. With the new data, only 7.6 +/- 4 trials were rejected. These details have now been clarified in the revised manuscript P10, L296-298.

P10.L24 Please provide the p-value for statistical test on trials removed between conditions.

Thank you for pointing out this valid point. To ensure that there was no significant differences between the amount of trials removed across conditions, the same repeated measures ANOVA was run on trials per condition across the remaining subjects, which showed no statistical significance: (n-back x noise interaction: $F_{1,18}$=0.9404, p=0.3450, n-back: $F_{1,18}$=0.0.0705, p=0.7937, noise: $F_{1,18}$=1.8331, p=0.1925). On average, for the remaining subjects, 8.10 +/- 1.37 trials remained in each condition. The statistics have now been added to the manuscript P10, L299-301.

P11.L2 Did all participants have a clear alpha peak (and not just a max)? Often I find that this is not the case, please state how you dealt with participants that did not have a clear alpha peak. There is some evidence that the IAF change with WM processing (Osaka et al, 1999 and Angelakis et al 2004). Is there a reason for estimating the IAF during the baseline and not the trial? Did the IAFs change between the baseline and the trial?

As discussed above, our analyses are no longer based on IAF. We did observe a large variation of the location of the alpha peak, but subjects generally showed a clear alpha peak. Only a single subject did not show a clear peak.

P11.L7 Using a 90% overlaps sounds like a lot. Was it considered to use smaller time-windows with less overlap? What was the reason for using a 90% overlap?

Thank you for this comment. Since we considered long trials with continuous speech, the power traces had more local fluctuations than what might be observed with shorter trials and more trial averages. Based on this, we find that overlapping windows provide a better picture of the oscillatory power changes over time. Note that the main statistical analyses were performed on the average over time, meaning that this redundancy did not affect the statistical results.

P11.L8 To me, it sounds like two different normalization procedures were used. In line 8 it sound like the average power for a particular condition was normalized by the average across all trials for each individual. In line 9, the topographies were normalized within each condition. Why this difference? As you show no 'raw' power topographies is this difference in normalization even necessary? Or are the topographies in Figure 3C not based on the same values as the timecourse powers in Figure 3A?

Thank you for pointing this out. We have changed this and the normalization procedure is now identical for the two analyses.

P11.L10 With the 90% overlap in the windowing procedure, the power was not strictly examined from 10-45 seconds as the 10-second measure of power contained information from 5 second and onwards, correct? What is the reason for disregarding the first 10 seconds of the trials? Any evoked response would be removed by disregarding the first ~.5 seconds.

We initially omitted the first 10 s of the trial because we use relatively long 3-digit numbers and it will thus take a while until the 1 vs 2 back tasks are differentiated. Our pupil and EEG power measures show transient effects at the beginning of the trial, likely related to the onset of the task, where items in WM gradually increase. We agree that disregarding the first 10 s may be unnecessary and we have changed the analysis to focus on 5-45 s.

P11.L19 Please specify which fixed value of the regularization parameter was used

The regularization parameter is now specified on P11, L326 ($\lambda=2^{12}$). In the new TRF analysis, we have optimized the regularization parameter to give the highest group-mean cross-validated prediction accuracy (in terms of Pearson's correlation) across all experimental conditions. We chose to use the same regularization parameter for all subjects to avoid differentially biasing the TRF amplitudes across subjects. In general, however, we find the prediction accuracies plateau over a large range of regularization parameters.

P11.L21 Please add (sampling frequency of 128 Hz) after the 7.8 ms.

This has now been added (P11, L327).

P12.L3 Is there any reason that you did not choose 12 electrodes, such as Di Liberto did? Is there any indication in you data that the same areas show good speech entrainment?

We have now changed the analysis to include 12 electrodes similarly to Di Liberto et al. (who used a 128-channel set). Regarding the last comment, a topographic plot of the entrainment effect has been included in the revised analysis (FIG4D), showing a good agreement with previous studies.

P12.L13 Was it the same three subjects that were removed from the EEG study?

The three subjects removed from the pupilometry data were different from the three subjects removed from the EEG power analysis. We have now specified this in the revised manuscript P12, L353-355.

P12.L16 Does the 200 ms baseline include the flash? A 200 ms baseline seems a little short compared to other pupilometry studies, can you please comment?

We have now clarified this in the revised manuscript. The 200 ms screen flash occurred before the period used as baseline. The flash served to measure the pupil light reflex, and the following black screen baseline to re-dilate the pupil. Following this, a grey screen was presented, to make the screen more comfortable for the participants to look at while performing the auditory task. Since the pupil constricts when changing from black to grey, only the last 200 ms period directly before the onset of the noise was used as baseline. In this way, the baseline was uncontaminated by constriction or dilation of the pupil, or by the influence of auditory stimulation. We agree that 200 ms is a relatively short baseline compared to other studies, however, we feel

that including either the onset of the noise or periods of pupil constriction would constitute a less reliable baseline. The details of the baseline definition are stated on P12, L356-358.

P12.L21 Please specify which software was used for the statistical tests

This has now been specified on P12, L364-365.

P12.L21 Where any of the ANOVAs checked for violation of the assumption of spherecity? If not, please do so and if necessary report the corrected p-values.

All statistical measures were checked for sphericity using the Shapiro-Wilk test. None of the statistical tests violated the assumption of sphericity.

Results

P13.L5 The non-significant effect of noise level was expected, correct?

This is correct. The higher noise level was set such that the speech should still be 100% intelligible (0 dB SNR). Therefore, it was assumed that this would not interact with the subjects' ability to solve the n-back task. A comment about this has now been added on P13, L381-382.

P13.L9 Is there any effect of noise level on the %correct over time?

As for the sensitivity, there was no effect of noise level over time on % correct.

P14.L20 As mentioned in the general comment it would be nice with analysis of a larger electrodespace and not just a single electrode (Fz or Oz). Especially the effect of theta, which is borderline significant would be strengthened by including more electrodes in the analysis.

We have conducted this analysis, as discussed above

P14.L21 A digit is missing in the p-value

This has been corrected, thank you.

P15.L14 How was the peaks and latencies of the TRF quantified (taking the maximum or visually determined)? And for which electrodes (average across the 6 chosen electrodes?).

Thank you for pointing this out. The peak of the TRF was identified by taking a maximum centered around the peak of the TRF from 100 ms to 300 ms. The latencies were then chosen as the point in time at which the TRF reached maximum amplitude. This was done at a subject-by-subject level on the average TRF response across the 12 chosen electrodes. A comment has been added to the manuscript on P11, L332-335.

P16.L5 Please specify the meaning of the sentence '..between the predicted response and the measured EEG'. Is the correlation not measured between the speech envelope and the speech envelope predicted from the EEG?

No, the 'forward mapping' approach employed here uses the envelope to predict the EEG response, similar as in e.g. Di Liberto et al. 2015, Power et al. 2012, or the Ding & Simon papers. This is unlike the 'backward mapping' or decoding approach (as in e.g. O'Sullivan et al. 2015) where an envelope is predicted from the multi-channel EEG. An advantage of the forward mapping, and our reason for taking this approach, is that it yields interpretable response functions (the TRFs) as a measure of the speech-evoked neural response.

P16.L14 It would be nice with a comment on why alpha power (8-15 Hz?) is analysed

Alpha entrainment is now analyzed from 8-13 Hz to be consisted with the frequency range used in the power analysis and previous studies. This has been corrected throughout the manuscript.

P16.L18 The results show a reduced correlation between the reconstructed speech and speech+noise, than for the speech alone, correct? If the noise is stationary with no envelope variation as stated previously in the

paper, how can this affect the correlation between the speech envelope and the predicted envelope? Could you please explain this result?

We did not examine a decoding model, i.e. we did not reconstruct the speech envelopes from the EEG responses. We compared correlations using either the envelope of the clean speech signal or the envelope of the speech + noise as predictors. Adding noise to the speech signal dramatically reduce the dynamic range of the envelope modulations, which can affect the TRF prediction accuracies. The description of our analysis procedure was not sufficiently clear, and we have updated this in the revised manuscript (P11, L315-346).

Discussion

P18.L4 The effects of alpha and theta power does not constitute a global change as stated when effects are only reported for single electrodes.

Correct, we have removed the term ‘global’.

P18.L5 I would refrain from calling it ‘auditory cortical theta-band activity’ , but use a more general term such as ‘activity in the area around auditory cortex’

We have changed this to the more generic ‘cortical activity’. We agree that we cannot make claims about the cortical origin of the entrained activity based on this data.

P18.L7 If indeed no significant effects are found in the delta-band activity, please delete the ‘largely’

Done.

P18.L8 Please add references to the figures in this paragraphs

References to figures have been included.

P18.L14 As previously stated, please take care when comparing results of auditory and visual experiments when it comes to alpha power

Please see our response to this point above.

P18.L18 The intermediate conclusion drawn in the last line of this paragraph does not seem appropriate.

This sentence has been removed.

P18.L22 Again, does a 1-minute trial really test a prolonged period of WM load

Please see our comment on this point above.

P18.L23 Although shown in Figure 3A, the result section does not describe any analysis of the change in EEG band power over the trial duration. Maybe a comment on the fact that alpha and theta are induced responses, while the pupil response is evoked would be appropriate in this context.

The relative change in theta and alpha over the trial duration is reported in the results. We find it difficult from the present results to conclude upon the evoked/induced nature of the observed pupil response as it evolves over the duration of the trial.

P18.L28 The pupil response is clearly larger for the 1-back than the 2-back task at time-point 0 s, but how is this anything other than the listeners ‘expected’ difficulty. There is no difference between the first number of the 1-back or the 2-back task, in both cases a cipher is presented and you have to remember it. If indeed the pupil size reflects effort/fatigue would it not be expected that the decrease in pupil size over time was larger for the easier task. It would be nice with some more comments on the pupil results and preferable some literature describing how the pupil dilation is expected to change over time.

The difference in pupil response between the 1-back and 2-back task is not present at time-point 0 s but only emerges after ~5 s. On average, this corresponds roughly to the onset of the 4th spoken number. The

1-back and 2-back tasks begin to become differentiated at this point, so we do not think that the differences can be ascribed to listeners' expected difficulty. If differences in expected difficulty were reflected in the pupil response then we would expect to see this effect at the onset of the trial. Effects related to effort and fatigue in the long-term evolution of the pupil response can be difficult to dissociate based on this paradigm as we discuss in the paper. We are not aware of any studies that have previously examined pupil responses at long durations comparable to the ones used in this study.

P19.L3 Again, the pattern is not global and you have not provided any statistical analysis of the EEG band power over time.

We have removed the term 'global' that was used in a wrong way, thank you.

P19.L9 I believe that Park et al looked at the phase coupling of alpha and not the band power

We now specify this (P19, L549-552).

P19.L12 For the sentence '…behavioural WM load during speech processing induces load-specific..', it could be argued that altering the noise level did not directly induce a behavioural WM load as no effect of noise was seen on d'.

It is correct that 'inhibition load' is not reflected in the behavioral performance, since the noise level was defined such that it does not affect speech intelligibility. Yet, listening to speech in noise at the border of intelligibility is arguably demanding. Other studies have shown effects of subjectively rated effort on speech-in-noise listening at 100% intelligibility (e.g. Wendt et al. 2016). It is a premise of our study design that the difference between the low and the high noise levels induces a load effect.

P19.L13 Please refer to figure 3 in the text

Done. (P19, L549)

P19.L13 Please refer to figure 4 in the text

Done. (P19, L548)

P19.L14 Please rewrite/specify the meaning of this sentence as is does not make sense. Also, please specify whether Park and Keitel investigate WM-dependent changes in the entrainment, or just coupling between delta-band activity and general speech entrainment.

We have rewritten this sentence. We hope that it is now clear that the studies of Park and Keitel only investigated the functional coupling and not WM-dependent changes (P19, L549-555).

P19.L21 Please refer to figure 3

Done. (P19, L557)

P19.L25 Ding and Simon does not find a general reduction in entrainment with more noise, they only observe a change for the most difficult SNR (-9 dB SNR). This finding indeed lead to the formulation of their theory on a 'gain-control mechanism' argued to cause this robust neural tracking of attended speech (Simon, 2015). A study by Petersen et al (2017) found effects of SNR level on the neural tracking of speech, but in elderly hearing-impaired listeners. It would be interesting with some comments on why you find SNR-effects with very positive SNR-values not leading to a performance difference, when Ding and Simon does not observe the same when using lower SNR-values which should affect the speech intelligibility.

Ding & Simon (2013) reported that the amplitude and latency of the M50TRF component, but not M100TRF component, change with increasing noise-level from -6 dB to +6 dB SNR. However, they did not include any data points for signal-to-noise ratios above 6 dB SNR in their regression analysis (due to nonuniformity on the regressor axis). In the present study, there was a 10 dB difference between the high noise level (0 dB SNR) and low noise (10 dB SNR) conditions. Although the regression analysis performed by Ding & Simon (2013) covers a stimulus SNR range of 10 dB, the perceptual difference between -6 dB and +6 dB SNR might be quite different from 10 dB to 0 dB SNR. In our study, the high noise level corresponds to a

considerable interference at the limit of intelligibility, while the noise at the low level is perceptually very faint. Moreover, it remains to be clarified why TRF profiles obtained with EEG, ECoG and MEG often differ in terms of latencies and amplitudes.

Doelling is missing from the reference list.

The missing reference has now been added to the reference list (P22, L652-654).

P19.L1
Be careful that you do not lead your readers to believe the selective attention (between two speech streams) and trying to search for/attend to a certain matching number in just one speech stream is the same thing. The attention that you refer to in the paragraph, the search of a match/mismatch is not something that has been investigated in relation to speech entrainment as far as I know. Although the speculation is good, there would be a way of analysing the speech entrainment to matching and mismatching targets to see if indeed there
is a difference in how they are encoded.

Thank you for this point. This interpretation is indeed a speculation. We have added the sentence: “Such a mechanism would need to be examined more closely, for example, by comparing entrainment to matching vs mismatching search targets.” (P18, L532-536)

.L8 Especially line 8 in the paragraph (‘In this case…’) confuses the two types of attention in my opinion.

Our speculation is that selective attention may be directed towards the phonological loop. We agree that this is not a view of selective attention that has been discussed previously. Yet, we find that this is a possible interpretation of our results that could be further tested in future work.

P19.L18 Please rewrite the sentence ‘The amplitude envelope is arguably…’. The amplitude envelope of what? A representation of ‘any acoustic signal …’, well not the signal without envelope variations. The term amplitude envelope is also used in line 22, please specify what it is an envelope of.

We have removed this part of the sentence (‘.. any acoustic signal’). We hope that other references to the envelope of speech signals are clear.

P20.L1 Although I’m not generally fond of the direct comparison between ERP and TRF, it should at least be specified what ERP-components the effect of background noise is found in and whether they present the background noise prior to the speech signal, as well as whether it was the level of the noise or the speech that was being manipulated. The results from Ding and Simon finds only background noise effects on the M50TRF component, but not on the component around M100TRF. This should be clearly stated.

We have rewritten this section (P18-19, L538-546). Since our new analysis did not reveal any effect of n-back on the TRF amplitudes we have removed the discussion of previous WM-related ERP studies. Regarding the background noise, we know specify that the N100 and P300 components were affected by noise in Whiting et al., 1998. As in our study, the background noise was present prior to the speech signals and the noise level was manipulated
We have pointed out that the effects of background noise were found on the M50TRF in the Ding and Simon reference.

P20.L15 There is something grammatically wrong with this part of the sentence

Thank you, we have fixed this.

P20.L17 ..and alpha entrainment. Please elaborate on why you think no effects of noise level were found on the performance or the pupil-response which should both be sensitive to WM load.

Previous studies have also reported effects of noise without an effect on performance or pupil-responses. Wendt et al. (2016), for example, found that higher noise levels (also set to yield full intelligibility) did not decrease performance but were subjectively rated as being more demanding. In that study, WM-demanding complex sentences yielded higher pupil dilations but the higher noise levels did not yield any dilations. In that study, a small effect of noise level on pupil dilations was found at the start of the trial, before the effect

of the sentence WM-task. This is similar to our current results where the n-back task seems to dominate the pattern of pupil dilations. It suggests that in paradigms with a WM task and an additional noise interference, pupil dilations are dominated by the effect of the task.

P20.L28 I'm missing a clear conclusion/concluding remark of the manuscript

We have expanded the initial section of the discussion to summarize the main findings (P16, L460-466).

P22 Abbreviations are missing the CDF

Thank you for pointing this out. CDF is now added to the abbreviation list (P21, L623-624).

P22 Besides the missing references, two of them contain …'s. Please correct this.

This has now been corrected.

Figure 1

Fig1 Figure 1 does not correspond to the description of the experimental procedure

We have revised the figure to include more details and hope that it now represents a correct overview of the experimental procedure.

Figure 2

Fig2A Is there a reason for using % correct in Figure 2A, while reporting d' in figure 2B? Consistency between the two figures is recommended.

Figure 2A displays hits as a function of time. Figure 2b displays the sensitivity over the trial duration which is the compound measure of hits and false alarms. We have revised Fig 2 in response to the comment below.

Why are the time-intervals overlapping? How is this possible, are answers from one time point counted in more than one time-interval? It's especially puzzling that the horizontal bars represent one standard deviation, so the actual overlap is even larger.

We have revised Figure 2. Now the figure displays the individual % correct data. The large circles represent the average performance for the respective first, second, third and fourth n-back targets in 1-back and 2-back contexts.

Fig2D The legend says peak dilation, while the plot is titles max dilation. Please be consistent. Legend Why are the performance (Fig 2A and 2B) giving error bars in standard deviation, while the error bars of the pupil measures are 1 SEM. Please be consistent or specify the reason for your choice.

Thank you for pointing this out. The legend and plot titles are now consistent. Furthermore, the error bars in figure 2A have now been changed to 1 SEM.

Please remove '* p<0.05, ** p<0.01' from the legend as it is not in the figure.

This has been implemented.

Figure 3

Fig3A It was not until seeing the figure that it was clear that you calculated relative power, please specify in the methods section.

The normalization procedure is specified on P10-11, L307-313. We have specified in the Methods section that we are computing the relative power.

Fig3C Please indicate the chosen electrodes in the topographic maps.

Done

Both the theta high-low noise effect and the 2-back – 1-back seem to have small effects around the relevant electrodes, could you please specify the size of the statistical effects just for comparisons.

The relevant statistical effects are specified in the Results section.

Legend Please delete ** and ***

Done.

2nd Editorial Decision 19 December 2017

Dear Dr. Hjortkjær,

Your revised manuscript was re-evaluated by the original two external reviewers and ourselves. We are pleased to inform you that we expect that it will be acceptable for publication in EJN following a few further minor revisions (please see attached comments of reviewer 2).

In your revision, please also attend to the following issues:
1. At this stage, please provide text and a figure file for the Graphical Abstract.
2. Your data sharing statement will need to be updated with further details (URL) now that the paper is to be accepted
3. Bar charts: lines representing the individual data points are too faint to see clearly. These won't print well.

If you are able to respond fully to the points raised, we shall be pleased to receive a revision of your paper within 30 days.

Thank you for submitting your work to EJN.

Kind regards,

John Foxe & Paul Bolam
co-Editors in Chief, EJN

Reviews:

Reviewer: 2 (Eline Borch Petersen, Eriksholm Research Centre, Denmark)

Comments to the Author
The revised analysis methods applied in the current version of the manuscript makes for a more readable and consistent paper. The authors work to include additional participants and change the analysis methods is appreciated and I only have a few additional comments to the current version of the manuscript.

1. In the comment made by the authors to my last concern (comment to P4.L10), they argue that they find consistent alpha patterns in studies employing auditory, visual, and motor tasks suggesting a more general role of posterior alpha. Although this might be the case, this does not justify completely disregarding the Functional Inhibition Theory put forward by Jensen and Mazaheri, 2007. In the introduction, the authors cite studies from the Obleser-lab all confirming the functional inhibition theory by showing increased occipital alpha with degraded auditory stimuli and increase WM load. However, visual rather than these auditory experiments are mentioned in the discussion. It would be nice with a justification of why studies with the same experimental task (n-back) across modalities is found more suitable to explain the observed effects than studies within the same modality, but with a slightly different experimental setup.

2. I still have some concerns on the number of channels used for the analysis of the oscillatory power. The conclusions regarding the alpha power is supported by the cluster-based statistics, however this analysis is not mentioned in the result section, which is should if it should be included. However, results for the cluster-based analysis of the theta activity are not mentioned at all. Looking at the topographic plot in Figure 3C top left, I would suspect that there would be a (non-significant?) cluster in this topographic area. If there is please report the p-value.

3. Please check the manuscript for correct usage of commas

Specific comments
P8.L229 P8.L238 Please specify that the green-screen flash is not including in Figure 1.

P10.L287 As the number of independent components vary with the number of bad/interpolated, it is sufficient to report only the percentage of removed components.

P10.L307+P17.L499 When normalizing to the average oscillatory across conditions, please be careful to not suggest that your findings are a sign of alpha desynchronization.

P11.L313 '...and all frequency bins', should this not be time bin

P13.L393 Please refer to Figure 2D after 'background noise'. Please specify the time-interval that the mean pupil dilation was averaged across

P14.L398- As mentioned in the general comment, please describe the results of the cluster-based analysis

P21.L620 Please add TFR to the list of abbreviations

Figure 2 The grey lines for each individual are difficulty to see, but a nice to have

Figure 2 caption Please specify what the shaded area of 2A illustrates. Please specify the 'over the trial duration' in seconds

Figure 3 In 3A, the dotted lines indicating the frequency interval are difficult to see (maybe whit lines are better). Maybe consider adding +/-1 SEM to the plots of relative alpha and theta power found under the TFRs

Figure 3 caption ...'(p>0.01, corrected)' the 'corrected' should be explained somewhere in the text or the caption.

Reviewer: 1 (Til Ole Bergmann, University of Tuebingen, Germany)

Comments to the Author
The authors addressed all of my previous concerns. I have no further comments.

Authors' Response 25 January 2018

Editors

1. At this stage, please provide text and a figure file for the Graphical Abstract.

Done.

2. Your data sharing statement will need to be updated with further details (URL) now that the paper is to be accepted

Done.

3. Bar charts: lines representing the individual data points are too faint to see clearly. These won't print well.

We have changed the lines to a darker color and hope they appear more clearly now.

Reviewer #2

Comments to the authors:

1. In the comment made by the authors to my last concern (comment to P4.L10), they argue that they find consistent alpha patterns in studies employing auditory, visual, and motor tasks suggesting a more general role of posterior alpha. Although this might be the case, this does not justify completely disregarding the Functional Inhibition Theory put forward by Jensen and Mazaheri, 2007. In the introduction, the authors cite studies from the Obleser-lab all confirming the functional inhibition theory by showing increased occipital alpha with degraded auditory stimuli and increase WM load. However, visual rather than these auditory experiments are mentioned in the discussion. It would be nice with a justification of why studies with the same experimental task (n-back) across modalities is found more suitable to explain the observed effects than studies within the same modality, but with a slightly different experimental setup.

As the reviewer points out and mentioned in the paper, a number of auditory studies have found a posterior *increase* in alpha power with noise interference or acoustic degradations. A number of studies (including auditory ones) have also reported alpha power to increase with working memory retention or load on WM storage (e.g. increasing alpha power with the number of items to be remembered). On the other hand, we observed a widespread *decrease* in alpha power with higher n-back level (and no effect of noise). Thus, functional inhibition of (task-irrelevant) visual areas does not appear to explain the observed changes in alpha power with n-back level. The widespread decrease in alpha power could reflect active processing in regions required for the task, as predicted by functional inhibition theory, but these would most likely not be auditory-specific. Instead, the observed alpha-theta power changes with n-back level are characteristically similar to those observed in visual tasks, suggesting the involvement of domain-general WM networks. We are not aware of auditory studies that demonstrate similar theta/alpha changes with the type of WM load that the n-back task targets.

2. I still have some concerns on the number of channels used for the analysis of the oscillatory power. The conclusions regarding the alpha power is supported by the cluster-based statistics, however this analysis is not mentioned in the result section, which is should if it should be included. However, results for the cluster-based analysis of the theta activity is not mentioned at all. Looking at the topographic plot in Figure 3C top left, I would suspect that there would be a (non-significant?) cluster in this topographic area. If there is please report the p-value.

We now report the cluster results in the results section. We did not find any significant clusters in the theta band at the given statistical threshold ($p<0.01$) and neighborhood extent (40 mm) (cf. Fig 3). The effect of n-back level on frontal theta was local, as also reported in previous n-back studies, and we recorded only 64 scalp electrodes. Also, when using fixed frequency bands to analyze theta power, the simultaneous widespread decrease in alpha power may partly limit the spatial extent of the effect in the group results. A more liberal statistical threshold could misleadingly suggest decreasing theta power at posterior electrodes, which is avoided by the *a priori* analysis.

3. Please check the manuscript for correct usage of commas

Done.

Specific comments

P8.L229+P8.L238 Please specify that the green-screen flash is not including in Figure 1.

Done.

P10.L287 As the number of independent components vary with the number of bad/interpolated, it is sufficient to report only the percentage of removed components.

Done.

P10.L307+ P17.L499 When normalizing to the average oscillatory across conditions, please be careful to not suggest that your findings are a sign of alpha desynchronization.

We have replaced the term 'desynchronization' and instead refer to 'task-related decrease in alpha power' P17.L510.

P11.L313 '...and all frequency bins', should this not be time bin

We have removed this to avoid confusion. The TFRs were normalized by the global power across time and frequency.

P13.L393 Please refer to Figure 2D after 'background noise'

Please specify the time-interval that the mean pupil dilation was averaged across

Done, thank you.

P14.L398- As mentioned in the general comment, please describe the results of the cluster-based analysis

Done.

P21.L620 Please add TFR to the list of abbreviations

Done.

Figure 2 The grey lines for each individual are difficulty to see, but a nice to have

We have changed the lines to a darker color.

Figure 2 caption Please specify what the shaded area of 2A illustrates

Please specify the 'over the trial duration' in seconds

Done. The trial duration varied depending on the speech list presented (~50 s).

Figure 3 In 3A, the dotted lines indicating the frequency interval are difficult to see (maybe whit lines are better)

Maybe consider adding +/-1 SEM to the plots of relative alpha and theta power found under the TFRs

We have changed the line color. The shaded areas in the relative alpha and theta power traces represent +/- 1 SEM.

Figure 3 caption ...'(p>0.01, corrected)' the 'corrected' should be explained somewhere in the text or the caption.

We have removed the term 'corrected' in the caption and added more details to the methods section about the correction procedure used in the cluster analysis (P13.L372).
